# Mechanistic insights into the rational design of masked antibodies

**DOI:** 10.1080/19420862.2022.2095701

**Published:** 2022-07-07

**Authors:** Carolina T. Orozco, Manuela Bersellini, Lorraine M. Irving, Wesley W. Howard, David Hargreaves, Paul W. A. Devine, Elise Siouve, Gareth J. Browne, Nicholas J. Bond, Jonathan J. Phillips, Peter Ravn, Sophie E. Jackson

**Affiliations:** a Yusuf Hamied Department of Chemistry, University of Cambridge, Cambridge, UK; b Biologics Engineering, R&D, AstraZeneca, Cambridge, UK; c Analytical Sciences, Biopharmaceutical Development, R&D, AstraZeneca, Cambridge, UK; d Analytical Sciences, Biopharmaceutical Development, R&D, AstraZeneca, Gaithersburg, MD, USA; e Discovery Sciences, R&D, AstraZeneca, Cambridge, UK; f Department of Chemical Engineering and Biotechnology, University of Cambridge, Cambridge, UK; g Living Systems Institute, University of Exeter, Exeter, UK; h Department of Biotherapeutic Discovery, H. Lundbeck A/S, Copenhagen, Denmark

**Keywords:** Masked antibodies, off-tumor cytotoxicity, protein design, pro-antibody, pro-drug, protein-protein interaction, pro-biologics

## Abstract

Although monoclonal antibodies have greatly improved cancer therapy, they can trigger side effects due to on-target, off-tumor toxicity. Over the past decade, strategies have emerged to successfully mask the antigen-binding site of antibodies, such that they are only activated at the relevant site, for example, after proteolytic cleavage. However, the methods for designing an ideal affinity-based mask and what parameters are important are not yet well understood. Here, we undertook mechanistic studies using three masks with different properties and identified four critical factors: binding site and affinity, as well as association and dissociation rate constants, which also played an important role. HDX-MS was used to identify the location of binding sites on the antibody, which were subsequently validated by obtaining a high-resolution crystal structure for one of the mask-antibody complexes. These findings will inform future designs of optimal affinity-based masks for antibodies and other therapeutic proteins.

## Introduction

Monoclonal antibodies have become a major class of therapeutics over the past twenty-five years and have proven to be promising as alternatives to, and often in combination with, conventional therapies for some cancers.^1–3^ Despite their clear potential, they can cause side effects, mainly due to on-target, off-tumor activity.^4^ In the past decade, strategies have emerged to improve the selectivity of therapeutic antibodies at the tissue level, by masking the paratope of antibodies, allowing them to be injected as a pro-drug (i.e., in an inactive form) that gets unmasked when they are in the target region.^5–7^ Two main categories of masks have been engineered so far: 1) masks with affinity for the antigen-binding domains, including peptides,^8–10^ antibody fragments^11–14^ and protein M^15^; and 2) non-binding masks that rely on steric hindrance to block the antigen-binding site. This second category includes coiled-coil domains,^16^ peptide-DNA assemblies,^17,18,19^ and a hinge region.^20^ Several different methods have been implemented to restore the activity of the masked antibody once it is at the target site.^21^ The most widely used approach has been activation by hydrolysis with enzymes that are overexpressed in the tumor or organ of interest.^9,10,11,12,13,16,15,20,22,23,24,25,26^ Alternatives have also been investigated,^21^ such as photo-cleavable linkers,^17^ pH-dependent peptide-DNA locks,^18^ or pH-sensitive masking peptides.^27^ A number of masked antibodies that effectively decrease binding to the target when not at the tumor site and improve therapeutic index (i.e., the ratio of maximal tolerated dose to therapeutic dose) have been successfully engineered. However, in the case of affinity masks, it is not yet well understood how to optimally design a masking moiety that has sufficient affinity to prevent the binding before activation, but that can efficiently dissociate after cleavage to allow binding to the target antigen.

The objective of our study was to understand what parameters affect the inactivation efficiency of a mask on an antibody, and how the relative affinities of the antibody for the mask *versus* the antigen dictate the level of activation required. We investigated the importance of difference parameters by engineering masked antibodies based on the well-characterized therapeutic antibody trastuzumab (Herceptin), which was used as a model. Trastuzumab is used to treat HER2-positive breast cancer, but it is known to provoke cardiotoxicity because some HER2 is expressed, albeit at lower levels, in cardiomyocytes.^28,29^ We fused anti-idiotypic antibody fragments, which had been previously developed as vaccines for breast cancer by mimicking HER2/NEU,^30,31^ to prevent binding to HER2, as well as an antibody fragment that was not specific to trastuzumab, to probe the effect of steric hindrance alone.

Through the use of anti-idiotypic antibody fragments masks composed of two structural domains, we were also able to assess the impact of a second protease cleavage site (previous studies having used only a single site within the linker) on the activation step.

At the start of the study, no high-resolution structural information was available on any of the trastuzumab/anti-idiotypic antibody fragment complexes selected. As location of the binding site of the mask was one of the parameters we wished to assess, state-of-the-art HDX-MS methodology was used to gain structural information. The structure of the complex formed between the antigen-binding fragment (Fab) of trastuzumab and the nanobody was solved during the course of the project, which also enabled us to confirm the ability of HDX-MS to accurately identify a binding site.

While other studies have been successful in masking antibodies and other therapeutic biologics (e.g., cytokines^32,33,34^), thereby establishing the strategy as a potentially powerful method of reducing off-target site activity, no clear rules for designing effective masks have been established. Here, by studying a series of masks with different affinities and association/dissociation rate constants, we establish the factors that are critical for both efficient masking and activation, thereby establishing the fundamental principles that underpin masking strategies in general.

## Results

### Design of a panel of masked antibodies with variable affinities

**Figure 1.**
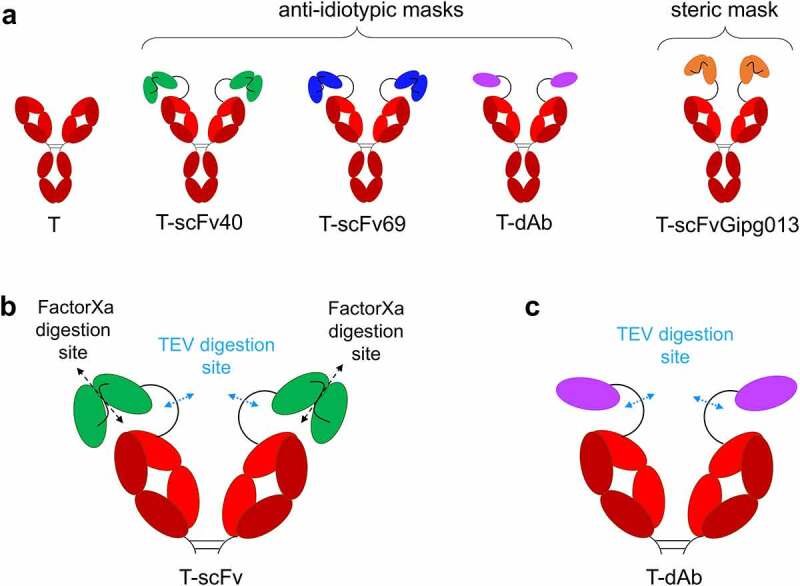
**Schematic of the antibodies engineered for this study and the location of the digestion site**(s). a) “naked” trastuzumab (T) serves as a positive control; T-scFv40, T-scFv69 and T-dAb are fusions of trastuzumab with various anti-idiotypic antibody fragments; T-scFvGipg013 is a fusion of trastuzumab with a scFv with no affinity for trastuzumab. b) Location of the digestion sites on the T-scFv constructs: TEV protease cleaves the linker between the antibody and the mask, and Factor Xa protease digests the linker between the two single-chain domains in the T-scFv constructs. c) Location of the digestion sites on T-dAb: TEV protease cleaves the linker between the antibody and the mask. For clarity, panels b and c show the Fab arms of trastuzumab only.

Three antibody fragments with affinity for trastuzumab were selected as anti-idiotypic masks, based on two single-chain variable fragments (scFv40 and scFv69) and one nanobody (dAb), previously developed and characterized by the Navarro-Teulon group.^30,31^ The three masks were successfully expressed and purified in isolation, as well as fusion proteins with trastuzumab to generate three masked antibodies (Figure 1a). An additional construct was also designed (T-scFvGipg013), using a scFv mask derived from the Gipg013 antibody, which targets the GIP receptor,^35^ to investigate the steric effects of a non-binding mask and to act as a control (Figure 1a). The antibodies with a single-chain mask were designed with two digestion sites: one is TEV protease-specific, located in the linker between the antibody and the mask, and the other is Factor Xa protease-specific, in the linker between the two single-chain domains (Figure 1b). The antibody fused with dAb just had a TEV digestion site in the linker (Figure 1c). The linker between the antibody and mask was the same for all three constructs and was 32 residues long (GSSGAGSGSAENLYFQGSGSAENLFQGSGGA, full sequences in **Table S2**). It should be stressed that the aim of the study was not to develop a new therapeutic antibody that would ultimately be tested in animal models and patients, but to design a tractable system to study the fundamental principles that underpin the strategy. As such, Factor Xa and TEV protease sites were selected in order to facilitate the *in vitro* studies undertaken. However, it would be necessary to modify the linker and include the sequence of tumor-specific enzyme for *in vivo* models. The relevance of tissue-specific enzymes for pro-drugs is discussed by Bleuez *et al*.^20^ All constructs were quality controlled by sodium dodecyl sulfate–polyacrylamide gel electrophoresis (SDS-PAGE) (***SI Material*, Figure S1**) and liquid chromatography mass spectrometry (LC-MS, **Figure S2** to **Figure S9, Table S1**). The sequences, including details of the linkers engineered between the trastuzumab and the mask, can be found in **Table S2**.

Figure 1 Alt text: Schematic of the different antibodies used, showing the antibody part (a Y-shaped protein), trastuzumab, in red, and the masks in different colors (scFv40 in green, scFv69 in blue, dAb in purple, scFvGipg013 in orange); the scFv masks are represented as two domains whereas the nanobody is just one. In panels b and c, the locations of the digestion sites are shown; TEV cleaves the linker between the antibody and the mask, and Factor Xa cleaves the linker between the two domains of scFv.

### Affinities of the different binding masks

**Figure 2.**
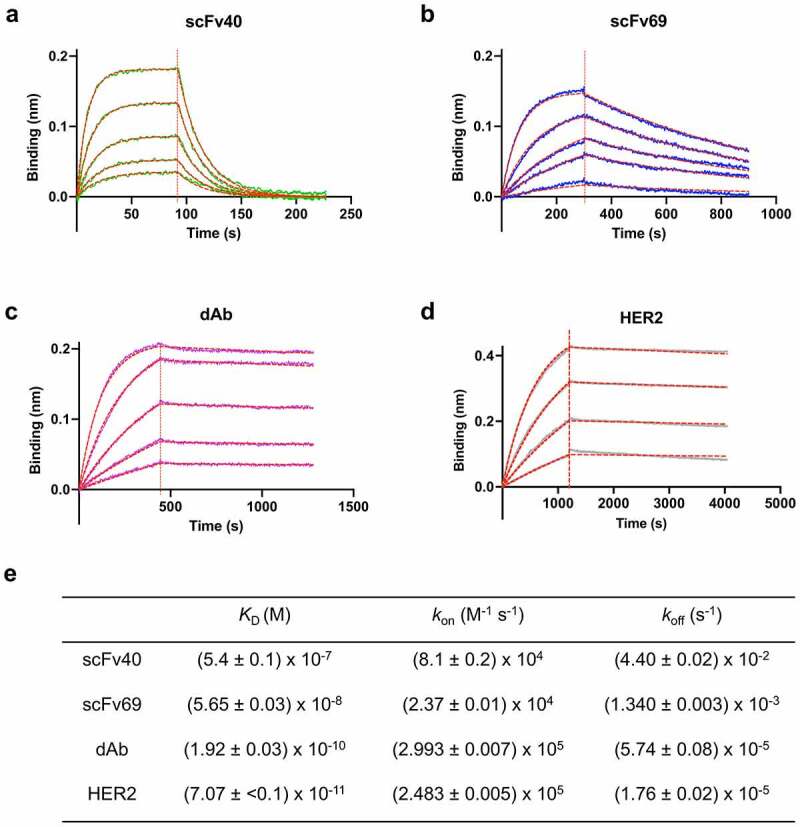
**Binding curves showing the association and dissociation of different masks to trastuzumab**. a) scFv40 (green: 46, 92, 184, 369, 738 nM). b) scFv69 (blue: 25, 100, 200, 300, 600 nM). c) dAb (purple: 1.7, 3.4, 6.9, 13.7, 27.5 nM). d) HER2 (gray: 0.9, 1.7, 3.4, 6.9 nM). In all cases, the dotted red line shows the best fit of the data to 1:1 binding model. e) Kinetic and thermodynamic parameters (*K*_D_, *k*_on_, *k*_off_) obtained from the best global fit of the association/dissociation data to a 1:1 binding model. The errors given are fitting errors from the global fitting.

The affinities of the anti-idiotypic antibody fragments scFv40, scFv69 and dAb for trastuzumab were measured by biolayer interferometry (BLI), using a range of concentrations for the masks. The data were globally fit to obtain high confidence values for the dissociation (*k*_off_) and association (*k*_on_) rate constants, and the ratio of the two, i.e., the equilibrium dissociation constant (*K*_D_), Figure 2e. The measured affinities of the masks for trastuzumab were 540 ± 10 nM for scFv40, 56.5 ± 0.03 nM for scFv69 and 0.192 ± 0.003 nM for dAb, which are lower (scFv40 and scFv69) or similar (dAb) to the affinity of HER2 for trastuzumab (*K*_D_ = 0.071 ± 0.001 nM). Although the exact values differ from those previously reported (likely due to the inversion of the V_H_ and V_L_ domains in our constructs compared with the originals), overall, the three antibody fragments showed the same trends,^30,31^ i.e., scFv40 has a large *k*_on_ but also a large *k*_off_, dAb has a large *k*_on_ and a very low *k*_off_, with values very similar to those of HER2 (Figure 2), and scFv69 has the lowest *k*_on_ while its *k*_off_ value is between those of scFv40 and dAb. The selected masks form a comprehensive panel with different affinities, ranging from 0.2 to 540 nM, as well as different association and dissociation rate constants.

Figure 2 Alt text: Figure made of five panels. In panels a to d, kinetic data, i.e., association and dissociation curves, are represented for the masks (scFv40, scFv69, dAb) and HER2. The stacked curves of four or five different concentrations for each mask or HER2 show an exponential increase in intensity for the association phase, and an exponential decrease in intensity for the dissociation phase. The fitting is represented in red in dashed lines. All association curves look similar, but the dissociation curves differ: for scFv40, the curves go back to 0 very quickly; for scFv69, the curves are less steep and do not all reach 0; for dAb and HER2, the curves are almost flat. The panel e is a table with the fittings of the curves.

## Efficiency of masks against HER2 binding while tethered to the antibody

The masking efficiency of scFv40, scFv69, dAb and scFvGipg013 fused to trastuzumab against HER2 was assessed using three different methods: 1) biolayer interferometry, where a fixed concentration of the biotinylated antigen HER2 was first bound to streptavidin biosensors, and subsequently the binding of the antibodies to HER2 was measured; 2) flow cytometry, where the binding of a serial dilution of antibodies to HER2, overexpressed on the surface of SK-BR-3 cells, was measured, and the half maximal effective concentration (EC_50_) extracted; 3) high content imaging (HCI) monitored by confocal microscopy, which shows the amount and the homogeneity of binding to the surface of SK-BR-3 cells.

**Figure 3.**
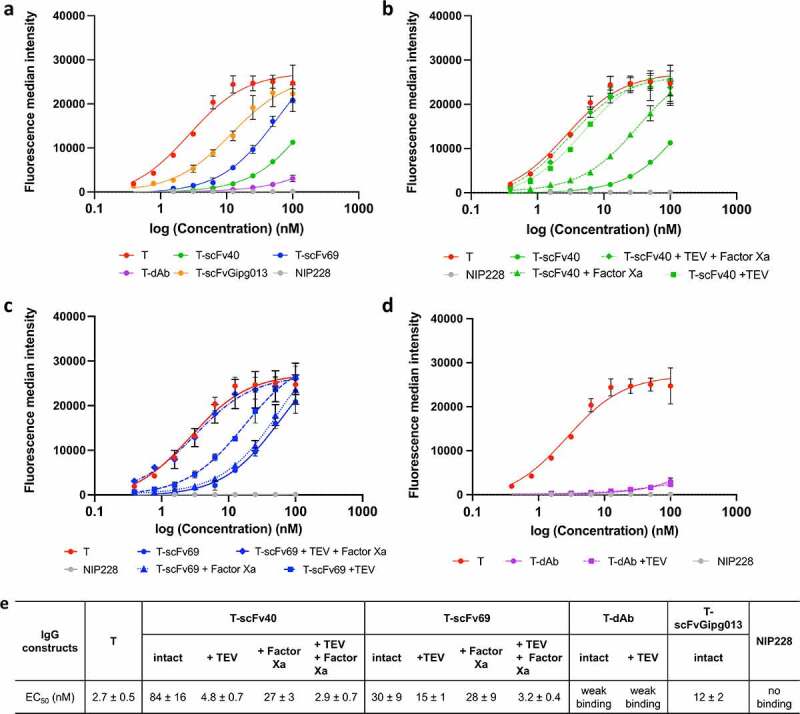
**Fluorescence median intensity measured by flow cytometry with channel detecting the AlexaFluor 647 fluorophore signal from a secondary antibody**. All experiments were performed in triplicate, the mean and the standard deviation are plotted. The data in panel a show the masking efficiency of the masked antibodies and control, whilst the data in panels b-d show the degree to which the masked antibodies can be activated by cleavage with proteases. a) Fluorescence median intensity of the secondary antibody showing the binding of the masked intact antibodies against HER2. b) Fluorescence median intensity of the secondary antibody showing the binding of the T-scFv40 constructs cleaved with TEV and/or Factor Xa to HER2, illustrating the degree of activation of the T-scFv40 upon cleavage. c) Fluorescence median intensity of the secondary antibody showing the binding of T-scFv69 to HER2 after cleavage with TEV and/or Factor Xa proteases. d) Fluorescence median intensity of the secondary antibody showing the binding of T-dAb to HER2 after cleavage of the linker with TEV protease. e) EC_50_ parameters obtained from the flow cytometry measurements, fitted to Equation 1.

**Figure 4.**
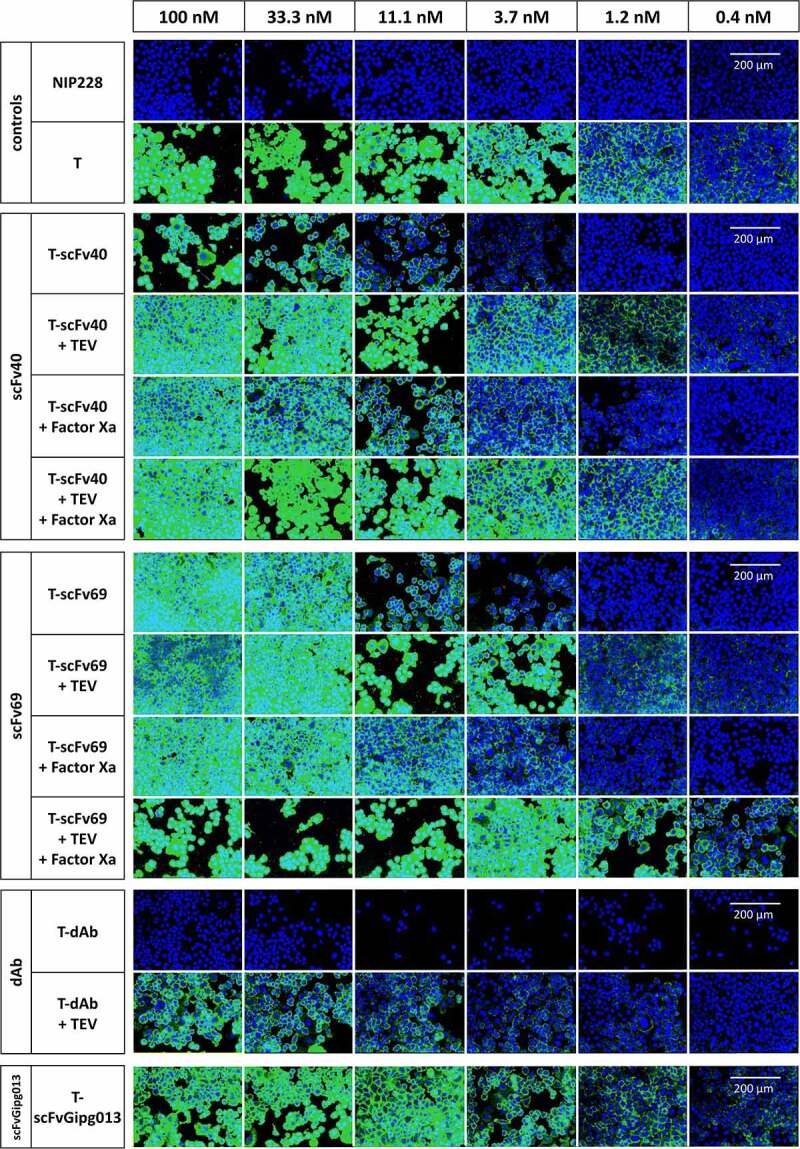
**Antibody binding to HER2 on SK-BR-3 cells, measured by immunofluorescence before and after cleavage of the linker between the mask and trastuzumab and between the two structural domains in the mask in the T-scFv40 and T-scFv69 constructs**. T alone is a positive control whilst NIP228 alone is a negative control; T-scFv40 and T-scFv69, intact, digested by TEV, Factor Xa, and both enzymes; T-dAb intact and digested by TEV; T-scFvGipg013 intact. All antibodies were detected with Alexa488 anti-human secondary (green) and nuclear Hoechst stain (blue).

The results from the three assays agreed, showing the same trends (Figure 3a, Figure 4, **Figure S10**). Naked trastuzumab had the strongest binding to HER2, i.e., the lowest EC_50_ of 2.7 ± 0.5 nM whilst T-scFvGipg013 significantly bound to HER2 although to a lower extent than naked trastuzumab. Its EC_50_ value was an order of magnitude higher (EC_50_: 12 ± 2 nM, Figure 3e), suggesting that the control scFv causes some steric effect, reducing the binding, but not suppressing it. Much lower binding to HER2 was observed for T-scFv40, T-scFv69 and T-dAb at the concentration used for BLI (12.5 µM, **Figure S10**), as well as for the lowest concentrations probed by flow cytometry and HCI (≤6.25 µM, Figure 3a, Figure 4), which highlights that affinity of the covalently linked antibody fragments for the antibody is necessary for effective masking.

Figure 3 Alt text: Figure made of five panels. In panels a to d, four graphs representing the binding (from fluorescence intensity) of intact and cleaved masked to HER2 on SK-BR-3 cells is shown as a function of antibody concentration. In panel a, the binding curves of intact antibodies to HER2 are shown: naked trastuzumab binds the most, followed by T-scFvGipg013, T-scFv69, T-scFv40, T-dAb and NIP228. In panel b, the binding of intact and cleaved T-scFv40 to HER2 is shown: naked trastuzumab, T-scFv40 + TEV and T-scFv40 + TEV+ Factor Xa bind the most and equally, followed by T-scFv40 + Factor Xa, followed by T-scFv40 intact. In panel c, the binding of intact and cleaved T-scFv69 to HER2 is shown: naked trastuzumab, T-scFv69 + TEV + Factor Xa bind the most and equally, followed by T-scFv69 + TEV, and followed by T-scFv69 intact and T-scFv69 + Factor Xa which bind equally. In panel d, the binding of intact and cleaved T-dAb to HER2 is shown: naked trastuzumab binds the most, T-dAb intact and cleaved bind equally and very little. The panel e is a table with the EC_50_ fittings of the curves.

Figure 4 Alt text: Large figure showing microscopy images of the binding to SK-BR-3 cells of the masked antibodies, intact and cleaved, at different concentrations. The bound antibodies are observed in green, and the cell nuclei in blue. For T-scFv40: T-scFv40 + TEV and T-scFv40 + TEV+ Factor Xa bind as much as naked trastuzumab, followed by T-scFv40 + Factor Xa, followed by T-scFv40 intact. For T-scFv69: T-scFv69 + TEV + Factor Xa bind as much as naked trastuzumab, followed by T-scFv69 + TEV, followed by T-scFv69 + Factor Xa which binds as much as T-scFv69 intact. For T-dAb: very little binding to cells is observed for the intact and digested constructs.

The flow cytometry and confocal microscopy provided further insight into the binding of the masked antibodies at higher concentrations. Although the anti-idiotypic masks strongly reduced the binding of trastuzumab to HER2, none of them totally prevented it, probably due to competition against an antigen with a higher affinity for the antibody. T-dAb showed the least binding to HER2 even at high concentrations (weak binding, Figure 3e), indicative of very effective masking, followed by T-scFv40 (EC_50_: 84 ± 16 nM, Figure 3e), and T-scFv69 (EC_50_: 30 ± 9 nM, Figure 3a, Figure 4**, Figure S10**).

It is interesting to note that the differences in masking capabilities do not directly correlate with the affinity of the mask for trastuzumab, as one might expect. For example, scFv40 prevents binding of trastuzumab to HER2 more effectively than scFv69, despite scFv69 having a stronger affinity for trastuzumab than scFv40 (Figure 3a). Instead, the results can be explained by kinetic factors. At all concentrations, there is a dynamic equilibrium between two different forms of the masked trastuzumab. In one form, the mask is sitting on top of the antigen-binding site, thus preventing it from binding to HER2; in the other, it has transiently dissociated from the antigen-binding site but remains covalently tethered through the linker^36^ (**Figure S11**). In this transient state, HER2 has the opportunity to bind. However, binding of HER2 is in direct competition with rebinding of the tethered mask. The degree of binding of HER2 therefore depends upon both the dissociation rate of the mask from trastuzumab and the relative association rates of the mask and HER2, which depend upon the association rate constants and the concentration of the species.

dAb has the highest affinity for trastuzumab and also shows the least binding to HER2. In this case, dAb has a very slow dissociation rate (*k*_off_ = (5.76 ± 0.08) x 10^−5^ s^−1^, Figure 2e), and therefore it comes off very rarely (half-life of dissociation is 3.3 hours), which does not provide many opportunities for HER2 to bind, especially over the time course of the incubation with SK-BR-3 cells. Additionally, dAb and HER2 have very similar association rate constants, which means that both proteins compete for trastuzumab when dAb comes off. Hence, HER2 binding is very low even at very high concentrations of T-dAb.

As previously mentioned, for scFv40 and scFv69, there is an inverse relationship between the affinity of the masks for trastuzumab and degree of binding of the masked trastuzumab molecules to HER2. This observation suggests that parameters other than affinity must come into play. We noticed a correlation between the *k*_on_ values and the EC_50_ values of the different masks, but not with *K*_D_ or *k*_off,_ indicating that the critical parameter may be *k*_on_. The correlation may derive from the following mechanistic rationale: the *k*_on_ for scFv40 is larger than for scFv69 (despite the weaker affinity of scFv40 for trastuzumab), meaning that an unmasked T-scFv40 will re-mask itself more quickly compared to an unmasked T-scFv69. Because T-scFv40 has a much shorter dwell time in the unmasked state, an unmasked T-scFv69 has a higher likelihood of encountering and binding to a HER2 molecule. Once the unmasked T-scFv69 binds a HER2 molecule, its tighter affinity for its mask is irrelevant because HER2 is now occupying the mask site and will likely not come off during the experimental time course as its dissociation rate is extremely slow (Figure 2). A schematic representation of this mechanism is illustrated in **Figure S11**. An alternative explanation to scFv69 being less effective than scFv40 in its ability to mask despite its higher affinity to trastuzumab is that it might not bind to the exact same location as scFv40 in the complementarity-determining regions (CDRs) of trastuzumab (see below in section: Investigation of the binding sites of trastuzumab to the masks).

In addition to providing information on the degree of binding, the confocal microscopy images showed that the binding was homogeneous across the surface of the cell membrane. Internalization was investigated over a short time course, which showed that trastuzumab did not internalize after 3 hours at 37°C in the SK-BR-3 cells (**Figure S12**), consistent with data previously published.^37^

Overall, all three masked antibody formats (T-dAb, T-scFv40, T-scFv69) showed significantly increased EC_50_ values compared to trastuzumab, and therefore meet the first requirement to decrease the binding of trastuzumab to HER2.

## Unmasking efficiency: HER2 binding measurements after digestion of the linker(s)

Following these observations, the activation of the antibody upon cleavage of the linker between the mask and trastuzumab or the linker between the two domains of the scFv masks was assessed. In particular, for the antibodies masked with scFvs, the aim is to understand if having two different cleavage sites, one between the antibody and the scFv, the other between the two domains of the scFv (Figure 1b), has a synergistic effect on activation. To evaluate this possibility, BLI, flow cytometry and high-content imaging were used following the same protocol as described previously, this time after digesting T-scFv40, T-scFv69 and T-dAb with TEV, Factor Xa, or both enzymes prior to the measurements. The completion of the digestion was verified by SDS-PAGE (**Figure S1**).

The results of the three assays showed that, for T-scFv40, cleaving the linker between the two scFv domains (Factor Xa digestion) only slightly increased its binding to HER2, but the digestion of the linker between antibody and the mask alone (TEV digestion) fully activated the antibody. As expected, cleaving both digestion sites also fully activated the antibody (Figure 3b, Figure 4**, Figure S10b**). In contrast, for T-scFv69, the cleavage of the linker between the two scFv domains by Factor Xa did not activate the antibody, whilst the digestion of the linker connecting the scFv to the antibody (TEV digestion) only partially activated the antibody. The digestion of both linkers, however, fully restored the binding of trastuzumab to HER2 (Figure 3c, Figure 4**, Figure S10c**). For T-dAb, cleavage of the linker between the antibody and the mask by TEV protease did not result in the activation of the antibody (Figure 3d, Figure 4**, Figure S10d**). These different levels of activation highlight the importance of the affinity of the mask for trastuzumab and the location of the digestion sites. scFv40 is the mask with the weakest affinity, in the micromolar range (Figure 2); covalently linking it to trastuzumab increases the EC_50_ by two orders of magnitude compared to naked trastuzumab for binding to HER2 on SK-BR-3 cells (Figure 3e). In this case, simply cleaving the linker between the antibody and the mask is sufficient to activate the antibody in the presence of HER2. This suggests two mechanisms. First, that the covalent linkage of scFv40 to the antibody increases the local concentration of the mask and therefore applies a stronger competition against HER2 for the variable domains of trastuzumab; and second, that in the absence of any covalent linkage between the antibody and the mask, trastuzumab preferentially binds to the very high-affinity antigen HER2 over the lower affinity svFv40. The scFv69 mask acts somewhat differently. The affinity of scFv69 for trastuzumab is stronger than that of scFv40, and, in particular, the dissociation constant of scFv69 is lower than that of scFv40. In this case, the fact that the digestion of the linker between the antibody and the mask is not sufficient to fully activate it shows that if the mask has a high affinity, hydrolysis of both digestion sites is required to fully activate the antibody. Finally, the dAb has a very similar affinity and dissociation constant to the antigen HER2 for trastuzumab, and therefore the cleavage of the linker has no effect as the mask effectively remains bound on the antibody and does not dissociate over the time course of the experiment.

Any potential effects of the presence of the TEV and Factor Xa enzymes were tested in a control flow cytometry experiment by measuring the binding of naked trastuzumab to HER2 in presence of TEV and/or Factor Xa enzymes. No difference in binding was observed (Figure S14).

## Interaction of the masks with trastuzumab after digestion of the linker

All the results presented previously were measured under conditions where there is competition between the mask and the antigen HER2 for trastuzumab binding. To understand to what extent the masks interact with the CDRs of trastuzumab after digestion, in the absence of competition with HER2, intact and digested samples were run on size-exclusion chromatography coupled with multi-angle light scattering (SEC-MALS).

SEC-MALS is a technique in which there is first a separation step by SEC, which provides a retention volume depending on the hydrodynamic radius of the species. This is followed by MALS, which can extract the species’ molecular weight (MW) when coupled to a concentration detector (refractive index or UV). The Fabs of all the masked antibodies (T, T-dAb, T-scFv40, T-scFv69, T-scFvGipg013) were generated to ensure the best resolution possible given the optimal MW range of the column and the protein standards used. Samples of intact Fabs were run alongside those digested by TEV and/or Factor Xa. Cleavage was verified by SDS-PAGE before the SEC-MALS experiment (**Figure S15**).

Consistent with the fact that the mask scFvGipg013 has no affinity for trastuzumab, the results of the TEV cleavage of the linker for T-scFvGipg013 are within the error of the naked trastuzumab Fab, showing that there is no nonspecific binding after cleavage (Table 1). For T-scFv40 Fab digested by TEV, two peaks, both with MWs close to that of the naked trastuzumab Fab, were observed. While both species have MWs close to that of naked Fab, the smaller of the two species is probably naked Fab whilst the slightly larger species eluting earlier might be the non-covalent Fab-svFv40 complex that dissociates during the chromatography step. The fact that scFv40 has high association and dissociation rates supports this hypothesis. The MW and retention time obtained for T-scFv40 Fab digested with Factor Xa show that the N-term of scFv40 (V_L_) that was cleaved is either still interacting with trastuzumab, or with the C-term of scFv40 (V_H_), similar to T-scFvGipg013. When T-scFv40 Fab is digested by both enzymes, only one peak is observed, and the MW measured is closest to naked trastuzumab. For T-scFv69 Fab, none of the digestions triggered the total unmasking of trastuzumab, as all MWs are within the error of the intact T-scFv69 Fab (Table 1), apart from a small peak observed with digestion by TEV alone and TEV combined with Factor Xa, which elutes close to naked trastuzumab (**Figure S16*c***). Finally, for T-dAb Fab, despite the digestion of the linker between the antibody and the mask, dAb still interacts with trastuzumab (Table 1**, Figure S16*d***). For all these experiments, the scFvs that were released were not observed because they are considerably smaller than the Fab and their signal too low to be quantitatively assessed.

**Table 1.** Molecular weights of intact and digested mask-Fab fusions measured by SEC-MALS.

	Refractive Index MW (kDa)
T	46 ± 2
T-dAb	61 ± 3
T-dAb + TEV	63 ± 3
T-scFv40	74 ± 4
T-scFv40 + TEV*	41 ± 2*
	47 ± 2*
T-scFv40 + Factor Xa	71 ± 4
T-scFv40 + TEV + Factor Xa	39 ± 2
T-scFv69	73 ± 4
T-scFv69 + TEV	68 ± 3
T-scFv69 + Factor Xa	71 ± 4
T-scFv69 + TEV + Factor Xa	67 ± 3
T-scFvGipg013	71 ± 4
T-scFvGipg013 + TEV	43 ± 2
T-scFvGipg013 + Factor Xa	53 ± 3
T-scFvGipg013 + TEV + Factor Xa	43 ± 2

The error values shown above are based on the average of fitting errors of duplicate measurements of BSA which gave an error of 5%. *Two peaks were observed for T-scFv40 cleaved with TEV.

Overall, these results show that the fate of the masks upon cleavage of the various linkers depends on the affinity of the mask for trastuzumab and, where relevant, the interactions between two domains of scFvs, the different masks remaining bound to different degrees to trastuzumab. Thus, activation of the masked antibodies by proteolytic cleavage relies on two factors, the *k*_off_ of the mask, and the competition between the anti-idiotypic antibody fragments and the targeted antigen.

## Investigation of the binding sites of trastuzumab to the masks

To measure the interaction surface experimentally in solution, hydrogen-deuterium exchange mass spectrometry (HDX-MS) was used. This technique is highly sensitive to the solvent accessibility and hydrogen-bonding of backbone amide groups in proteins, and has been used successfully to investigate interactions of antibody domains.^38,39^

**Figure 5.**
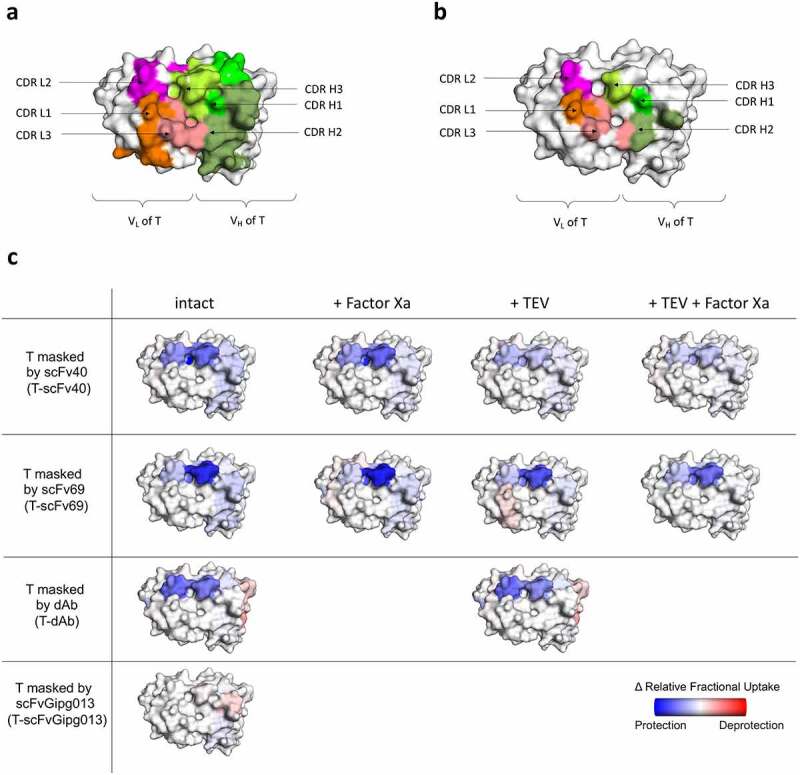
**Schematic of the results of the HDX-MS experiments superimposed on the structure of trastuzumab**. Crystal structure (1N8Z) of the variable domains of trastuzumab observed from the top. Shown in color and labeled are the CDRs of both the LC and HC showing the possible binding sites. a) CDRs of the V_L_ and V_H_ of trastuzumab. b) Paratope of trastuzumab when interacting with HER2, generated by highlighting the residues which have atoms that are within 4 Å of HER2; the CDRs L3 and H3 are the main contributors to the binding of HER2 according to Cho et al., 2003.^40^ c) Relative change in fractional deuterium exchange represented as a color scale: reduced exchange (blue), no change (white), increased exchange (red), for trastuzumab interacting with the different masks scFv40, scFv69, dAb and scFvGipg013, with the linkers intact or digested by TEV and/or Factor Xa.

The first objective was to identify the binding sites of the masks to trastuzumab when they are covalently linked by probing the differences in the protection of amide protons in the CDRs of trastuzumab. The crystal structure of HER2 in complex with trastuzumab Fab solved by Cho *et al*.^40^ showed that HER2 mainly interacts with CDR3 in both the light chain (LC) and heavy chain (HC) (CDRs L3 and H3). The HDX-MS data acquired showed that the highest levels of protection, when the masks are covalently linked to trastuzumab, were observed on CDR L2 and the CDR H3, while the other CDRs did not show a large degree of protection (Figure 5). More specifically, scFv40 and dAb provided substantial coverage of both CDR L2 and CDR H3 of trastuzumab, while scFv69 primarily covers CDR H3 and only minimally occluded the CDR L2 (Figure 5). As expected, scFvGipg013, which does not have affinity for trastuzumab, did not provide any protection to any of trastuzumab’s CDRs (Figure 5, **Figure S17**). The flow cytometry data showed that scFv69 was less effective than scFv40 in reducing the binding of trastuzumab to HER2, despite having a higher affinity (Figure 3). The HDX-MS data support the hypothesis that scFv69 covers less well the paratope of trastuzumab than scFv40 (Figure 5), and therefore inactivates the antibody less efficiently, although this finding does not exclude the possible role, proposed earlier, of the importance of the kinetic parameters. The HDX-MS results suggest that the binding to both CDR L2 and CDR H3 may be more effective in preventing the binding of HER2 to trastuzumab than the coverage of CDR L2 alone, which explains why scFv69 is not an ideal mask against HER2, despite its high affinity. This observation highlights the importance of the binding location to generate an effective mask, but it is interesting to note that the masks are effective despite the fact that they do not cover the whole of the HER2 paratope. The epitope coverage of the masks was also measured with HDX-MS, and is represented in Figure S18.

Figure 5 Alt text: Paratope of trastuzumab observed from the top. Panel a shows the paratope and the CDRs of the heavy and light chains are colored. Panel b shows the paratope, where the residues that interact with HER2 are colored. Panel c is a representation of the protected regions on the paratope, in blue, according to the HDX-MS results, for each of the masks (scFv40, scFv69, dAb and scFvGipg013), for intact and cleaved molecules. T-scFv69 has a smaller surface of protected regions compared to scFv40 and dAb, and the cleaved molecules still show blue regions on the paratope, although lighter.

**Figure 6.**
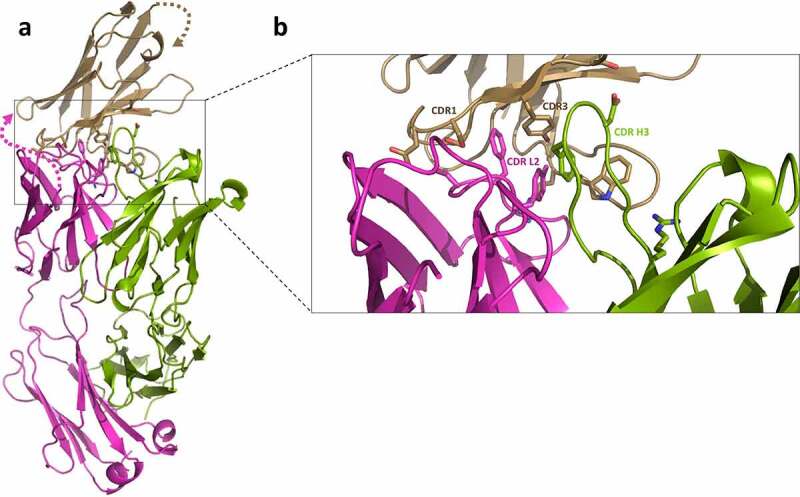
**Crystal structure of T-dAb showing the interactions between trastuzumab variable Fab fusion and the dAb domain (PDB deposition 7PKL)**. a) Shows the overall fold of the protein. Trastuzumab heavy chain is shown in green, the light chain in magenta and the fusion dAb in wheat. Key side residue:side chains interactions are shown as sticks. The 32-residue linker between the trastuzumab light chain and the dAb was not visible in the electron density (marked with dotted lines). b) Close up view of key residues in the CDR. CDR H1, H2, L1 and L3 are not involved in the interaction (not labeled). The bulk of the interaction is derived from trastuzumab CDR L2 and CDR H3 side chain interactions with dAb CDR1 and CDR3.

To elucidate which specific regions of the dAb and trastuzumab contribute to the measured affinities and HDX-MS protection, a crystal structure was determined at 2.35 Å resolution (Figure 6). The model shows a single T-dAb Fab fusion in the asymmetric unit. Examination of the interface reveals the main dAb:T Fab interactions are located in CDR L2 and CDR H3 on trastuzumab and CDR1 and CDR3 in the dAb. CDR H1, CDR H2, CDR L1 and CDR L3 of trastuzumab are not involved in the interaction (Figure 6b). The co-ordinates were used to calculate the buried surface area of the trastuzumab-dAb interface using PISA,^41^ which was 405.8 Å^2^ and the complex (without the linker) was predicted to be stable in solution. Therefore, the X-ray structure confirms the observations from the HDX-MS experiments that show that dAb mainly protects the CDR L2 and CDR H3 of trastuzumab from exchange (Figure 5c, T-dAb intact).

Figure 6 Alt text: Crystal structure of trastuzumab linked with dAb, with a zoomed-in window of the side chains of the residues interacting at the interface between the antibody and the mask.

## Investigation of the deprotection of the CDRs upon cleavage of the linkers

The second objective of the HDX-MS experiments was to determine how the protection levels in trastuzumab and the masked domains changed after digestion, in the absence of HER2. For trastuzumab masked by scFv40, cleaving the linker between the two scFv domains by Factor Xa showed the same level of protection in CDR L2 and CDR H3. The cleavage of the linker between the antibody and the mask by TEV protease, or the cleavage of both linkers by TEV and Factor Xa, resulted in less protection than in the intact construct (Figure 5c, Figure 1b**, Figure S17**), consistent with the mask at least partially dissociating from trastuzumab. It is worth noting that even after cleavage by TEV and Factor Xa, some protection relative to naked trastuzumab was still observed showing that the mask still interacts transiently with trastuzumab’s CDRs, under the conditions used, corroborating the SEC-MALS experiments. These results demonstrate that the covalent linkage of scFv40 to trastuzumab effectively increases the local concentration of the mask, which therefore increases the percentage bound, hence its protection.

When trastuzumab is masked by scFv69, the CDR H3 is afforded extensive protection even when the linkers are cleaved by either TEV or Factor Xa. Only the combination of TEV and Factor Xa gave rise to a lower level of protection in CDR H3 (Figure 5c). Finally, when trastuzumab is masked by dAb, the digestion did not affect the protection, consistent with other results that strongly suggest that the dAb remains bound to trastuzumab under the conditions used due to its high affinity and very slow dissociation rate.

## Discussion

In this study, we investigated the properties that affect the efficiency of an anti-idiotypic antibody fragment mask to 1) reduce the antibody binding to its antigen when it is not at its target site, and 2) enable effective activation of the antibody upon cleavage of the linker(s) at the target site. We successfully engineered three masked antibodies based on trastuzumab with covalently linked anti-idiotypic antibody fragments,^30,31^ which were shown to be successful at preventing HER2 binding to different degrees.

The results on the intact constructs provide information on how well the masks cover the antigen-binding region of trastuzumab and the location of its binding interface, as well as establishing their effectiveness in inactivating the antibody. The flow cytometry and microscopy results showed that dAb is the most effective mask, followed by scFv40, and finally scFv69. As this order does not simply follow that of the relative affinities of the different masks for trastuzumab, other factors must also be involved. One possible factor is purely structural: the HDX-MS results showed that scFv40 and dAb both clearly cover the CDR H3 and the CDR L2 in trastuzumab, whereas scFv69 primarily protects the CDR H3. From structural studies by Cho *et al.*,^40^ it is known that HER2 interacts mainly with CDR3s of the HC and LC. This highlights the fact that for scFv40 and dAb, binding to CDR L2 in addition to CDR H3 may result in steric hindrance and blocking of binding of HER2 to CDR L3, thus leading to them being more effective masks. The other possible factor is kinetic: the BLI measurements reveal that dissociated scFv40 reassociates, and therefore remasks, trastuzumab faster than scFv69, as indicated by the difference in their *k*_on_ values. Therefore, a transiently dissociated T-scFv69 gives HER2 a wider time window in which to capture the unmasked trastuzumab. In contrast, after dissociating, scFv40 gives HER2 much less time to capture trastuzumab in its unmasked state. The EC_50_ results from the HER2 binding assays for the various masks correlate with their *k*_on_ values, and not with their affinities or *k*_off_ values, supporting this hypothesis. Therefore, how effectively a covalently-linked mask inactivates an antibody and increases the EC_50_ depends on its binding site and its association kinetics, compared to those of the antigen.

The EC_50_ values show that scFv69 increases the EC_50_ by 11-fold, scFv40 by 31-fold and dAb by an even higher factor (Figure 3e), similar to what has been observed in other studies. For example, Desnoyers *et al*.^8^ engineered a probody, consisting of an EGFR-targeting antibody tethered to a short peptide mask, which increased the EC_50_ by 47-fold. Lu and Chuang *et al*.^25^ engineered a pro-infliximab rheumatoid arthritis therapy for higher selectivity and safety, where the mask reduced the antigen-binding affinity by 395-fold. Chen *et al*.^22^ designed a EGFR antibody masked by a latency-associated peptide whose EC_50_ was boosted by 7.5-fold.

In order to be useful as a therapeutic agent, effective masking of the antigen-binding site must be combined with efficient activation of the antibody when it reaches its target. Thus, the cleavage and subsequent dissociation of the mask is as important as efficient initial masking. Our results show that both affinity and dissociation rate are critical in the activation process. Results from the BLI, flow cytometry and microscopy experiments show that, for T-scFv40, cleaving the linker between the antibody and the mask is sufficient to activate the antibody, whereas for T-scFv69, the cleavage of both linkers is necessary for full activation. This observation agrees with the results from the SEC-MALS and HDX-MS experiments, which show that, after cleavage, scFv69 interacts more strongly with trastuzumab than scFv40. The fact that the non-covalently bound scFv40 and scFv69 still transiently bind to trastuzumab in the absence of HER2, does not prevent the binding of trastuzumab to HER2 in its presence. This reflects that the activation of the antibody is driven by both the affinity and dissociation kinetics of the mask. Additional evidence of this is seen with dAb, for which cleavage did not lead to activation over any relevant timescale because its *k*_off_ is orders of magnitude less than those of the other two masks. In this case, there is no opportunity for competition between the mask and the antigen because the mask does not dissociate over relevant timescales. The relative affinities of antigen and mask may also influence activation: HER2 has a much stronger affinity than either scFv40 or scFv69, which were both successfully activated. In contrast, masks with affinities close to that of the antigen, such as the dAb, may not be so useful, because the mask may not dissociate over suitable timescales.

Therefore, we hypothesise that to facilitate activation of the antibody, 1) its value of *k*_off_ cannot be too low and needs to be higher than the antigens, and 2) the antigen needs to be able to bind to antibody faster than the cleaved and dissociated mask rebinds, suggesting the *k*_on_ value for the mask may need to be lower than the antigen. These observations for *k*_off_ and *k*_on_ indicate that the mask most likely needs a higher *K*_D_ and therefore lower affinity compared to the antigen.

Our study evaluated the impact of three different anti-idiotypic antibody fragment masks on the HER2-binding activity of trastuzumab. The results provide valuable information that will guide future design of antibodies and other therapeutic proteins that can be effectively inactivated by a covalently tethered affinity-based mask, and efficiently activated by protease cleavage. We demonstrate that the combination of activity assays, such as flow cytometry, and biophysical characterization with HDX-MS, forms a very powerful strategy for assessing the suitability of any potential mask. Moreover, the agreement observed in this study between HDX-MS and X-ray crystallography illustrates how HDX-MS can be leveraged as a convenient substitute allowing an initial analysis of whether a mask may be effective, and used to characterize the molecular interactions important to stabilize mask-antibody complex. HDX-MS not only provides information on the paratope coverage of trastuzumab, but also on the epitope within the masks, which allows rational protein engineering, for example, by targeting residues in the mask’s CDRs that are involved in the protein-protein interaction.

**Figure 7.**
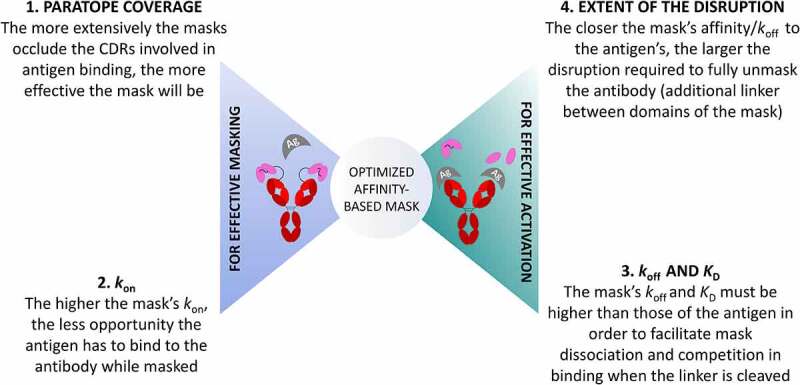
**Summarized representation of the proposed four key factors contributing interdependently to the optimal design of an anti-idiotypic mask**: two define the effective masking of an antibody to avoid on-target off-tumor effects, and two others influence the effective activation of the masked antibody upon proteolytic cleavage.

Taken together, this work reveals that four major parameters need to be optimized to successfully engineer an effective affinity-based mask (Figure 7). First, the mask must bind to either the same paratope as the antigen or a nearby site that effectively blocks binding of the antigen. Second, the higher the *k*_on_ of the mask, the less opportunity the antigen has to bind to the antibody when it is masked; however, it needs to be lower than the antigen’s *k*_o__n_ to enable it to bind to the antibody when the mask is released. Third, the mask’s *k*_off_ and *K*_D_ must not be lower than or the same order of magnitude as those of the antigen to favor the antigen binding by competition with the mask after cleavage. Fourth, higher affinity masks can be used if the interaction between the mask and the antibody can be disrupted more, through the cleavage of linkers not only located between the mask and the antibody, but also within the domains of the mask.

Figure 7 Alt. text: Schematic showing on the left side the two parameters dictating effective masking (1. Paratope coverage: The more extensively the masks occlude the CDRs involved in antigen binding, the more effective the mask will be. 2. *k*_on_: the higher the mask’s *k*_on_, the less opportunity the antigen has to bind to the antibody while masked), and on the right side, the two parameters dictating effective unmasking (3. *k*_off_ and *K*_D_: The mask’s *k*_off_ and *K*_D_ must be higher than those of the antigen in order to facilitate mask dissociation and competition in binding when the linker is cleaved; 4. Extent of disruption: The closer the mask’s affinity/*k*_off_ to the antigen, the larger the disruption required to fully unmask the antibody (additional linker between domains of the mask))

Our results comprise an important basis for understanding the parameters that can play a role in determining the efficiency of masking and activation of a pro-drug *in vitro*, and a framework that can be used to consider and interpret data from both *in vitro* and *in vivo* studies. The fact that we have shown that association and dissociation kinetics can play a role in addition to affinity is significant, and therefore kinetics should be considered in any project involving the design of masked antibodies and other therapeutic proteins. Further work is, of course, necessary in order to test whether these parameters are also important *in vivo*. We believe they may, and we therefore anticipate our conclusions can be applied broadly to any affinity masking strategies, whether it be for therapeutic biologics or some other application.

## Materials and methods

### Design and expression of masked antibodies and antibody fragments

The three anti-idiotypic antibody fragments investigated in this study were based on scFv40, scFv69 and a llama single domain, previously developed by Navarro-Teulon’s group.^30,31^ Here, the order of the domains was inverted for scFv40 and scFv69, such that the N-terminal V_L_ domain was fused to the C-terminal V_H_ domain. An additional construct was also designed with a control scFv, scFvGipg013, with the sequence of the variable domains of Gipg013,^35^ which targets the GIP receptor and does not bind to trastuzumab, to investigate the steric effects. The DNA strings encoding for scFv40, scFv69 and dAb were synthesized (GeneArt) and cloned as fusions to either a His-tag, for expression of the masks, or to the trastuzumab LC^40^ for expression of the masked antibodies (sequences in **Table S2**), using standard molecular biology techniques. The vectors used were proprietary mammalian expression vectors. Successful cloning was confirmed by Sanger sequencing (SourceBioscience Sequencing). The antibody fragments scFv40, scFv69, dAb and the full-length antibody fusions T-scFv40, T-scFv69, T-dAb, T-scFvGipg013 were transiently expressed in Chinese hamster ovary (CHO) cells using proprietary medium.^42^ The His-tagged proteins, scFv40, scFv69, and dAb, were purified from culture supernatants using HisTrap™ Excel purification columns (Cytiva) equilibrated with Dulbecco’s phosphate-buffered saline (DPBS) (pH 7.4). An initial wash with DPBS and 20 mM imidazole pH 7.4 was performed to remove any nonspecific binding, and then the proteins were eluted with DPBS supplemented with 500 mM imidazole pH 7.4. Finally, the proteins were buffer exchanged into DPBS pH 7.4 using PD-10 desalting columns (Cytiva). For T, T-scFv40, T-scFv69, T-dAb, T-scFvGipg013, the cleared culture supernatant was loaded onto a MabSelect SuRe column (Cytiva) preequilibrated with DPBS pH 7.4. The proteins were eluted with 0.1 M glycine pH 2.7, and buffer exchanged into DPBS pH 7.4 using PD-10 desalting columns (Cytiva).

The purity of the proteins was verified by SDS-PAGE and the verification of correct MW was achieved by LC-MS analysis (**Figure S2** to **Figure S9**). For full-length IgGs, glycosylated reduced samples were generated by reducing 50 µg with 1 µL of 500 mM DTT for 30 min at 37°C. For all proteins, 1 µg was injected onto the column. The liquid chromatography desalting step was carried out on a reverse phase column (Acquity UPLC® BEH300 C4 1.7 μm 2.1 × 50 mm column, Waters Part #186004495), using an aqueous phase A (H_2_O, 0.01% trifluoroacetic acid (TFA) and 0.1% formic acid) and an organic phase B (acetonitrile with 0.01% TFA and 0.1% formic acid), running at 0.15 mL/min. For the reduced method, 95% phase A was run for 3 minutes, followed by a one-minute gradient from 95 to 75% of phase A, then a 10-minute gradient from 75 to 55% phase A, and finally a one-minute gradient from 55 to 20% A (and maintained at 20% phase A for another minute). For the intact method, 95% phase was run for 1 min, followed by a 2-minute gradient from 95 to 20% A, and then by a 3-minute gradient from 20 to 5% B. The mass was analyzed either on the mass spectrometer Synapt G2 (Waters) or Q Exactive Orbitrap (ThermoFisher). Synapt G2 was operated in a positive polarity under sensitivity mode, with a capillary voltage of 3.4 kV, source temperature 120°C, sampling cone at 60 V, desolvation temperature of 400°C and desolvation gas flow of 800 L/hour. Q Exactive Orbitrap was operated in a positive polarity. For both the intact method and the reduced methods, MS data were acquired over a 500–4500 m/z range, using an AGC target of 3e6 and a maximum injection time of 200 ms, with a resolution of 15000. The capillary voltage was 3.5 kV, capillary temperature was 300°C, auxiliary gas heater temperature was 250°C. The intact method was run with a funnel radio frequency (RF) level of 100, in-source CID of 50 eV, and the reduced method was run with a funnel RF level of 70 and in-source CID of 80 eV. Data acquired on the Synapt G2 was analyzed on MassLynx and that acquired on the Q Exactive Orbitrap was analyzed on BioPharmaFinder.

For SEC-MALS and X-ray crystallography, the full-length antibodies were digested by the enzyme FabALACTICA® (Genovis, Sweden), a protein that cleaves human IgG1 antibodies at a specific site above the hinge (… KSCDKT/HTCPPCP …) and generates two single Fab arms and the Fc domain with hinge. The digestion was carried out overnight at 37°C, using 2000 units to digest 2 mg of antibody. The digested Fab fragments were then purified with a CaptureSelect™ CH1-XL column (Life Technologies, ThermoFisher, Netherlands), run in DPBS and eluted with 0.1 M glycine pH 2.7. The recovered Fab domain pool was further purified by on SEC chromatography with DPBS (Superdex® 200 Increase 10/300 GL, Cytiva) to separate the single-armed Fabs from some undigested antibody. The purified Fabs were verified by SDS-PAGE (**Figure S15**).

Both the full-length IgG and the Fab domains of the antibody fusions were digested with TEV and Factor Xa, without further purification prior to the activity assays and the biophysical experiments. The TEV protease digestion (TEV protease T4455-1KU, Sigma-Aldrich) was carried out using 1 unit of enzyme for 20 µg of substrate overnight at 30°C. The Factor Xa digestions (Factor Xa protease, New England Biolabs, catalog number: P8010S) were performed with 1 µg of enzyme per 50 µg of antibody, incubating the samples for 3 hours at 37°C. The completion of the digestion was verified by SDS-PAGE.

### Measurement of the affinity of the masks (scFv40, scFv69 and dAb) for trastuzumab by biolayer interferometry

The affinity of the masks scFv40, scFv69 and dAb for trastuzumab was measured by BLI with the OctetRED384 (ForteBio). All samples were prepared in DPBS (Sigma-Aldrich) with 0.1% bovine serum albumin (BSA) solution, 30% BSA in DPBS, sterile-filtered, BioXtra, Sigma-Aldrich) and 0.02% Tween 20 (TWEEN® 20 for molecular biology, Sigma-Aldrich). Serial dilutions were prepared for each of them: for scFv40, 46, 92, 184, 369, 738 nM; for scFv69, 25, 100, 200, 300, 600 nM; for dAb, 1.7, 3.4, 6.9, 13.7, 27.5 nM. Anti-human IgG Fc Capture (AHC) Biosensors (ForteBio) were used to immobilize trastuzumab at 5 µg/mL. Before starting the BLI experiment, the biosensors were incubated in DPBS with 0.1% BSA and 0.02% Tween 20 for 10 min. For the measurements, the biosensors were first incubated for one minute in DPBS with 0.1% BSA and 0.02% Tween 20 for the first baseline, trastuzumab was then loaded onto the biosensors until reaching a displacement of 0.8–1 nm, the biosensors were then incubated in DPBS with 0.1% BSA and 0.02% Tween 20 for the second baseline for one minute, and subsequently moved to wells containing the serial dilutions of the different masks for the association step, and finally moved to DPBS with 0.1% BSA and 0.02% Tween 20 for the dissociation step. The association and dissociation results were fit to a 1:1 model using a global fitting.

### Measurement of the binding of the intact and digested masked antibodies for HER2 by biolayer interferometry

The binding of masked antibodies, intact and digested, to HER2 was measured by BLI. The antibodies T-scFv40, T-scFv69 were digested with either TEV or Factor Xa, or both enzymes together. T-dAb was only digested by TEV. The effective digestion was verified by SDS-PAGE prior to the BLI experiments (Figure S1). T, T-scFv40, T-scFv69, T-scFvGipg013, NIP228^43^ (used as a negative control) were used in their intact form. All samples were prepared at 2.5 µg mL^−1^ in DPBS with 0.1% BSA and 0.02% Tween 20. Biotinylated HER2 (Her2/ERBB2 Protein, Human, Recombinant (His & AVI Tag), Biotinylated, Sino Biological, catalog number: 10004-H27H-B) was prepared at 5 µg mL^−1^ in DPBS with 0.1% BSA and 0.02% Tween 20.

Streptavidin (SA) Biosensors (ForteBio) were used to immobilize the biotinylated HER2. The assay was carried out with the following steps: Baseline 1: incubation in DPBS with 0.1% BSA and 0.02% Tween 20 for 1 min; Loading: incubation in solution of biotinylated HER2 for binding to the biosensors for 1 min; Baseline 2: incubation of the biosensors in DPBS with 0.1% BSA and 0.02% Tween 20 for 1 min; Association: incubation of the biosensors with solutions containing intact and digested masked antibodies for 300 s; Dissociation: incubation of the biosensors in DPBS with 0.1% BSA and 0.02% Tween 20 for 300 s.

### Measurement of the binding of the intact and digested masked antibodies for HER2 expressed on SK-BR-3 cells by flow cytometry

SK-BR-3 cells, breast cancer cells overexpressing HER2, were grown in culture. At passage number 8 and 80% confluency the cells were harvested using standard accutase (Gibco) treatment. The cells were washed with DMEM (Gibco) 1% fetal bovine serum (FBS, heat inactivated, Gibco) 1% Pen Strep antibiotic (Penicillin-Streptomycin (10,000 U/mL), Gibco) and resuspended in DPBS 2% BSA. 100,000 cells/well were dispensed in 96-well plates (PP V-bottom, Chimney Well, natural, Greiner Bio-one International). The intact antibodies T, T-scFv40, T-scFv69, T-dAb, T-scFvGipg013, NIP228, as well as the digested antibodies T-scFv40 + TEV, T-scFv40 + Factor Xa, T-scFv40 + TEV + Factor Xa, T-dAb + TEV (and T + T EV, T + Factor Xa, T + TEV + Factor Xa as controls, **Figure S14, Table S3**) were diluted to concentrations ranging from 0.4 to 1600 nM in DPBS 2% BSA, and incubated with the SK-BR-3 cells for 30 min at 4°C. The cells were then washed with DPBS 2% BSA once, resuspended in 100 µL, and the secondary antibody Alexa Fluor® 647 anti-human IgG Fc (BioLegend, catalog number: 410713) was added to the cells at 0.5 μL/100,000 cells for 30 min at 4°C. The cells were washed once with DPBS 2% BSA and fixed with 4% paraformaldehyde in DPBS for 1 hour at room temperature. Finally, one additional washing step was performed prior to resuspension in DPBS 2% BSA. All experiments were conducted in triplicate.

The binding of the various antibodies was detected monitoring the fluorescence of the secondary antibody Alexa Fluor® 647 anti-human IgG Fc (BioLegend, catalog number: 410713) using a BD LSRFortessa™ Flow Cytometer. The first gate (P1) selected singlets, based on the forward scatter height (FSC-H: forward scatter height) and the forward scatter area (FSC-A: forward scatter area). The second gate (P2) selected the viable cells according to the side scatter area (SSC-A) and the FSC-A. Subsequently, the fluorescence intensity of APC (channel to detect Alexa 647) was measured.

Some hook effect was observed at high concentrations (full raw data is presented in **Figure S14**). This can be caused by excessively high concentrations of the primary antibody simultaneously saturating both HER2 and the secondary antibodies. The high-dose hook effect occurs mostly (but not exclusively) in one-step immunometric (sandwich) assays, giving a decrease in signal at very high concentration of primary antibody.^44,45,46^ Therefore, the signal obtained is probably caused by insufficient washes between the incubation of the primary and secondary antibodies. The higher concentration points were therefore excluded from the fitting as they are not representative of the actual binding to HER2 and are artifacts of the protocol followed. The other data points were fitted to an agonist *versus* response fit using GraphPad Prism 9 and Equation 1:

(1) $$Y\;=\;a+\;X^{*}\left(b\;-\;a\right)/\left(EC_{50}\;+\;X\right)$$

Where a and b are the upper and lower plateaux of the curves, X the concentration of antibody and EC_50_ the half maximal effective concentration.

The parameters used for the flow cytometer can be found in **Figure S13**.

### Measurement of the binding of the intact and digested masked antibodies for HER2 expressed on SK-BR-3 cells by confocal microscopy

SK-BR-3 cells were plated at 2 × 10^4^ per well of 96-well plate (CellCarrier-96 Ultra Microplates, PDL-coated, black, 96-well, Perkin Elmer) in DMEM and were incubated overnight at 37°C. The next day, cells were incubated with primary antibodies for 30 min at 4°C: intact antibodies T, T-scFv40, T-scFv69, T-dAb, T-scFvGipg013, NIP228, as well as the digested antibodies T-scFv40 + TEV, T-scFv40 + Factor Xa, T-scFv40 + TEV + Factor Xa, T-dAb + TEV were diluted to concentrations 0.4, 1.2, 3.7, 11.1, 33.3, 100 nM in HBSS (Hank’s Balanced Salt Solution, Gibco) with 1% FBS. After washing to remove excess antibody, cells were fixed with 4% paraformaldehyde for one hour at room temperature. The cells were then permeabilized and stained by incubation with a mixture of HCS CellMask® Orange Stain (ThermoFisher) diluted to 1:25000, Hoechst stain (33342, Trihydrochloride, Trihydrate, ThermoFisher) diluted to 1:5000 and 0.1% Triton (Sigma-Aldrich) in HBSS 1% FBS and incubated for 30 min at room temperature. After washing, the secondary antibody Alexa Fluor® 488 goat anti-Human IgG (ThermoFisher, catalog number: A-11013) was added to the cells for 30 min at room temperature (60 µL at 2 µg/mL per well), then washed and imaged. All experiments were done in duplicate and one representative image was shown for each condition.

High-throughput confocal microscopy was performed using the Opera (PerkinElmer) with filters, and exposure times according to the manufacturer’s instructions using 20X water objective. The same contrast was used for all images.

### Size-exclusion chromatography coupled to multi-angle light scattering (SEC-MALS)

For the masked antibodies, the Fab arms obtained from the FabALACTICA digestion of full-length IgG were used in order to achieve higher resolution in the SEC-MALS experiment. Samples were prepared in PBS pH 7.4 at 0.25–0.5 mg/mL, and ~ 1–20 ug were injected onto a column equilibrated and run in PBS pH 7.4. The SEC column was a TSKgel® G3000SWXL HPLC Column phase diol, L × I.D. 30 cm × 7.8 mm, 5 μm particle size (Tosoh Bioscience, Germany). The experiment was conducted using Agilent Technologies 1260/1290 Infinity and Wyatt DAWN-Optilab instruments. The refractive index and multi-angle light scattering data were analyzed using the Astra software version 7.3.2.

### Hydrogen-deuterium exchange mass spectrometry (HDX-MS)

The HDX-MS experiments were conducted on full-length IgGs T, T-scFv40, T-scFv69, T-dAb, T-scFvGipg013 both intact and digested by TEV and/or Factor Xa, except for T-scFvGipg013. The peptide map (**Figure S19**) was generated in an equilibration buffer of 10 mM potassium phosphate pH 7.5 in H_2_O for T, using a data-dependent acquisition (DDA) MS^2^ approach. MS data were acquired using an Orbitrap Fusion (ThermoFisher) over a 300–2000 m/z range, using an AGC target of 200000 and a maximum injection time of 100 ms. MS^2^ was acquired in the ion trap in centroid mode, using an AGC target of 10000 and a maximum injection time of 35 ms. Fragmentation was achieved by higher-energy C-trap dissociation using a collision energy of 30%. The labeled data on the twelve samples were recorded after 50, 500 and 5000 s of incubation at 20°C in deuterated buffer (10 mM potassium phosphate pD 7.5 in D_2_O) with an MS^1^ method (300–2000 m/z range, AGC target of 200000 and maximum injection time of 100 ms). All experiments were run in triplicate.

On the Leap robot, 7 µL of 10 µM protein in equilibration buffer (10 mM potassium phosphate pH 7.5 in H_2_O) was diluted 7-fold at 20°C into equilibration or labeling buffer (10 mM potassium phosphate pD 7.5 in D_2_O), to generate a peptide map or labeled data, respectively. The mixture was then diluted 1:1 with quench buffer at 4°C (100 mM potassium phosphate, 8 M urea, 0.5 M TCEP, pH 2.5). The quench solution was then injected into a Waters nanoAcquity UPLC system, flowing for 4 min at 400 µL/min onto the pepsin column at 20°C for digestion (Waters Enzymate™ BEH Pepsin Column (2.1 x 30 mm, 5 μm)) to the trap (pushed by LC-MS grade H_2_O with 0.2% formic acid), and then eluting from the C18 analytical column for 10 minutes, using a 5% to 40% organic phase (acetonitrile with 0.2% formic acid).

A peptide map was generated using BioPharmaFinder from equilibration data (10 mM potassium phosphate pH 7.5 in H_2_O) acquired with a MS^2^ method. Around the N-linked glycosylation in the C_H_2 domain, the peptides including the asparagine at position 297 (in Eu numbering) were expected to carry the most abundant glycosylation expressed in CHO cells, i.e., G0F. The peptides obtained were filtered by confidence score higher than 80%. The exported csv file with the peptides as well as that same non-deuterated data were imported into HDExaminer, to operate a second filtration of the peptides; only the charge state with the highest intensity from the peptide map data was kept per peptide. After the peptide pool was curated, the labeled data were added, and the D incorporation per peptide data was then exported as a csv file. To identify which deuterium incorporations were significant and to observe the deuterium exchange for each time point separately and overall, the data processing method used was first described by Dobson, Devine, Phillips *et al*.^38^ Starting with a csv file containing the D incorporation data, this Matlab-coded method first assesses if the incorporation of deuterium per peptide is significant compared to the control sample (assessed by a t-test, if two-tailed p-value < 0.01), then sums the significant time points per peptide, subsequently converts the peptide D incorporation to amino-acid incorporation by dividing the D incorporation by the maximal number of D that can be exchanged per peptide, subtracting the D incorporation from the control and dividing the amino-acid incorporation by the redundancy, and finally normalizing it. The output is the deuterium incorporation for each individual amino acid. The crystal structure of the Fab domain of trastuzumab (PDB:1N8Z) was colored with a red-white-blue scale according to the relative incorporation of deuterium per amino acid. The summary HDX-MS parameters are shown in **Table S4**, the peptide map in **Figure S19**, and the full uptake plot for the light and heavy chains are presented in **Figure S20** and **Figure S21**, respectively.

### X-ray crystallography

Crystals of T-dAb Fab were grown by sitting drop vapor diffusion. The crystallization reagent was 0.2 M ammonium sulfate, 0.1 M sodium acetate (pH 4.6) and 25% w/v PEG 4000. 200 nL of the protein solution of T-dAb Fab was mixed with 200 nL of the reagent and equilibrated over a reagent well containing 50 µl at 20°C. Crystals with a rod-like morphology (100 µm) appeared in the drops after 13 days. Crystals were cryo protected using 23% butane-2,3 diol added to the well solution and snap frozen in liquid nitrogen. X-ray data were collected on beamline IO4 at the Diamond Light Source. Data processing was performed using autoPROC^47^ and STARANISO (Global Phasing Ltd) and showed acceptable data extending to 2.35 Å. Molecular replacement was achieved using PHASER^48^ and a trastuzumab model (PDB 4HJG) with a nanobody model (PDB 6DBE). Coot,^49^ Refmac^50^ and Buster^51^ (Global Phasing Ltd) were used during model building and refinement. The final model was in space group P 2_1_ 2_1_ 2 with cell dimensions of a = 160 Å.7, b = 59.9 Å, c = 63.5 Å α = β = γ = 90° and showed a single T-dAb Fab fusion in the asymmetric unit. Data collection, processing, refinement, and model statistics can be found in **Table S5**.

## Abbreviations

BLI (biolayer interferometry); BSA (bovine serum albumin); CDR (complementary determining region); CHO (Chinese hamster ovary); CID (collision-induced dissociation); D (deuterium); dAb (domain antibody); DNA (deoxy ribonucleic acid); DPBS (Dulbecco’s phosphate-buffered salt solution); EC50 (half maximal effective concentration); FBS (fetal bovine serum); HBSS (Hanks balanced salt solution); FSC (forward scattered area); HC (heavy chain); HCI (high content imaging); HDX-MS (hydrogen-deuterium exchange mass spectrometry); LC (light chain); LC-MS (liquid chromatography mass spectrometry); MS (mass spectrometry); MW (molecular weight); PEG (polyethylene glycol); RF (radio frequency); scFv (single-chain variable fragment); SDS-PAGE (sodium dodecyl sulfate polyacrylamide gel electrophoresis); SEC-MALS (size-exclusion chromatography – multi-angle light scattering); T (trastuzamab); V_H_ (variable heavy chain); V_L_ (variable light chain).

## Supplementary Material

Supplemental Material
