# Supplementary material for: Mechanistic insights into the rational design of masked antibodies
Source: MAbs. 2022 Jul 7;14(1):2095701. doi: 10.1080/19420862.2022.2095701 (PMC9272835; doi:10.1080/19420862.2022.2095701)
Supplement: Supplemental Material [file KMAB_A_2095701_SM2653.docx]

**SUPPLEMENTAL INFORMATION**

**Figure S1:** **SDS-PAGE of the purified antibodies and scFvs and verification of the digestion of the masked antibodies (IgG) with TEV and/or Factor Xa protease for biolayer interferometry, flow cytometry, microscopy and HDX-MS experiments.** T: trastuzumab; T-dAb: dAb fused to trastuzumab on LC; T-scFv40 : scFv40 fused to trastuzumab on LC; T-scFv69 : scFv69 fused to trastuzumab on LC. L: Ladder: Novex® Sharp pre-stained protein standard (ThermoFisher).

**Figure S1 Alt text:** Picture of a SDS-PAGE, where the position of the bands show the molecular weight of the protein. The ladder is on the left hand-side, and fourteen samples were analyzed, each corresponding to one column. T-scFv40 and T-scFv69 show the main band at 200 kDa, T-dAb at 175 kDa, and when digested by TEV and Factor Xa, the main band migrated further (decreased further) and smaller bands corresponding to the digested fragment appeared.

**Figure S2:** **Verification of the correct molecular weight of trastuzumab IgG by LC-MS in its glycosylated reduced form** (chromatogram, lock mass corrected combined spectrum and deconvoluted mass). Mass spectrometry data acquired on a Synapt G2 (Waters). The comparison between the theoretical and experimental masses can be found in **Table S1**. The deconvoluted mass of the heavy chain corresponded to the sum of the protein molecular weight and the glycosylation G0F (shown in the top right-hand corner).

**Figure S2 Alt text:** Liquid chromatography mass spectrometry figure made of three panels to quality-control check trastuzumab. In the first panel, the chromatogram is represented, showing two peaks, one corresponding to the heavy chain and the other to the light chain. In the second panel, the combined spectra of the heavy and light chains are shown. In the third panel, the deconvoluted mass of the glycosylated heavy (50620 Da) and light (23441 Da) chains are shown.

**Figure S3:** **Verification of the correct molecular weight of trastuzumab IgG masked by scFv40 (T-scFv40) by LC-MS in its glycosylated reduced form** (chromatogram, combined spectrum and deconvoluted mass). Mass spectrometry data acquired on a Q Exactive Orbitrap (ThermoFisher). The comparison between the theoretical and experimental masses can be found in **Table S1**. The deconvoluted mass of the heavy chain corresponded to the sum of the protein molecular weight and the glycosylation G0F (shown in the top right-hand corner).

**Figure S3 Alt text:** Liquid chromatography mass spectrometry figure made of three panels to quality-control check T-scFv40. In the first panel, the chromatogram is represented, showing two peaks, one corresponding to the heavy chain and the other to the light chain. In the second panel, the combined spectra of the heavy and light chains are shown. In the third panel, the deconvoluted mass of the glycosylated heavy chain (50619.86 Da) and light chain fused to scFv40 (52011.59 Da) are shown.

**Figure S4:** **Verification of the correct molecular weight of trastuzumab IgG masked by scFv69 (T-scFv69) by LC-MS in its glycosylated reduced form** (chromatogram, combined spectrum and deconvoluted mass). Mass spectrometry data acquired on a Q Exactive Orbitrap (ThermoFisher). The comparison between the theoretical and experimental masses can be found in **Table S1**. The deconvoluted mass of the heavy chain corresponded to the sum of the protein molecular weight and the glycosylation G0F (shown in the top right-hand corner).

**Figure S4 Alt text:** Liquid chromatography mass spectrometry figure made of three panels to quality-control check T-scFv69. In the first panel, the chromatogram is represented, showing two peaks, one corresponding to the heavy chain and the other to the light chain. In the second panel, the combined spectra of the heavy and light chains are shown. In the third panel, the deconvoluted mass of the glycosylated heavy chain (50619.72 Da) and light chain fused to scFv69 (51943.40 Da) are shown.

**Figure S5:** **Verification of the correct molecular weight of trastuzumab IgG masked by dAb (T-dAb) by LC-MS** **in its glycosylated reduced form** (chromatogram, combined spectr*um* and deconvoluted mass). Mass spectrometry data acquired on a Synapt G2 (Waters). The comparison between the theoretical and experimental masses can be found in **Table S1**. The deconvoluted mass of the heavy chain corresponded to the sum of the protein molecular weight and the glycosylation G0F (shown in the top right-hand corner).

**Figure S5 Alt text:** Liquid chromatography mass spectrometry figure made of three panels to quality-control check T-dAb. In the first panel, the chromatogram is represented, showing two peaks, one corresponding to the heavy chain and the other to the light chain. In the second panel, the combined spectra of the heavy and light chains are shown. In the third panel, the deconvoluted mass of the glycosylated heavy chain (50625 Da) and light chain fused to dAb (39696 Da) are shown.

**

**Figure S6:** **Verification of the correct molecular weight of trastuzumab IgG masked by scFvGipg013 (T-scFvGipg013) by LC-MS in its glycosylated reduced form** (chromatogram, combined spectra and deconvoluted mass). Mass spectrometry data acquired on a QExactive Orbitrap (ThermoFisher). The comparison between the theoretical and experimental masses can be found in **Table S1**. The deconvoluted mass of the heavy chain corresponded to the sum of the protein molecular weight and the glycosylation G0F (shown in the top right-hand corner).

**Figure S6 Alt text:** Liquid chromatography mass spectrometry figure made of three panels to quality-control check T-scFvGipg013. In the first panel, the chromatogram is represented, showing two peaks, one corresponding to the heavy chain and the other to the light chain. In the second panel, the combined spectra of the heavy and light chains are shown. In the third panel, the deconvoluted mass of the glycosylated heavy chain (50619.71 Da) and light chain fused to scFv69 (51931.01 Da) are shown.

**Figure S7:** **Verification of the correct molecular weight of scFv40 by LC-MS (combined spectrum and deconvoluted mass)**. Mass spectrometry data acquired on a Synapt G2 (ThermoFisher). The comparison between the theoretical and experimental masses can be found in **Table S1**.

**Figure S7 Alt text:** Liquid chromatography mass spectrometry figure made of two panels to quality-control check scFv40. In the first panel, the combined spectrum of scFv40 is shown. In the second panel, the deconvoluted mass of scFv40 is shown (27116 Da).

**Figure S8:** **Verification of the correct molecular weight of scFv69 by LC-MS (combined spectrum and deconvoluted mass).** Mass spectrometry data acquired on a Q Exactive Orbitrap (ThermoFisher). The comparison between the theoretical and experimental masses can be found in **Table S1**.

**Figure S8 Alt text:** Liquid chromatography mass spectrometry figure made of two panels to quality-control check scFv69. In the first panel, the combined spectrum of scFv69 is shown. In the second panel, the deconvoluted mass of scFv69 is shown (27046.8 Da).

**Figure S9:** **Verification of the correct molecular weight of dAb by LC-MS (combined spectrum and deconvoluted mass).** Mass spectrometry data acquired on a Q Exactive Orbitrap (ThermoFisher). The comparison between the theoretical and experimental masses can be found in **Table S1**.

**Figure S9 Alt text:** Liquid chromatography mass spectrometry figure made of two panels to quality-control check dAb. In the first panel, the combined spectrum of dAb is shown. In the second panel, the deconvoluted mass of dAb is shown (14572.36 Da).

**Figure S10: Biolayer interferometry: measurement of the wavelength shift due to binding of the trastuzumab constructs to HER2.** The first half of the time course up to 500 s corresponds to the association phase, whilst the second half corresponds to the dissociation phase. (A) Binding of the intact covalently linked masked antibodies and controls to HER2. (B) Binding of T-scFv40 after cleavage of the linker(s) with TEV and/or Factor Xa to HER2. Shows activation efficiency. (C) Binding of T-scFv69 after cleavage of the linker(s) with TEV and/or Factor Xa to HER2. Shows activation efficiency. (D) Binding of T-dAb after cleavage of the linker against with TEV to HER2. A shows the masking efficacy whilst B-D show the activation efficiency.

**Figure S10 Alt text:** Figure made of four panels, showing four graphs representing the binding (of intact and cleaved) masked to HER2 as a function of antibody concentration, using biolayer interferometry. In panel a, the binding curves of intact antibodies to HER2 are shown: naked trastuzumab binds the most, followed by T-scFvGipg013, T-scFv69, T-scFv40, T-dAb and NIP228. In panel b, the binding of intact and cleaved T-scFv40 to HER2 is shown: naked trastuzumab, T-scFv40 + TEV and T-scFv40 + TEV+ Factor Xa bind the most and equally, followed by T-scFv40 + Factor Xa, followed by T-scFv40 intact. In panel c, the binding of intact and cleaved T-scFv69 to HER2 is shown: naked trastuzumab, T-scFv69 + TEV + Factor Xa bind the most and equally, followed by T-scFv69 + TEV, and followed by T-scFv69 intact and T-scFv69 + Factor Xa which bind equally. In panel d, the binding of intact and cleaved T-dAb to HER2 is shown: naked trastuzumab binds the most, T-dAb intact and cleaved bind equally and very little.

**Figure S11: Schematic representation of the effect of k_on_ of the mask on the binding of HER2 to the transiently dissociated trastuzumab-mask complex:** an ideal mask should have a $k_{on}^{mask}$ which is lower than the antigen’s, so it can bind when the linker is cleaved and the mask is unbound, but it shouldn’t be too low so that when it is tethered, the mask can compete with the antigen and prevent it from binding.

**Figure S11 Alt text:** Schematic representing three masked antibodies in different states (where the mask is covalently to the antibody), with arrows between them. The first schematic represents the masked antibody with the mask on, the second schematic represented the masked antibody with the mask off (though still covalently linked), and the third schematic represents the masked antibody with the mask off (still covalently linked) and the antigen binding to the variable domains. There is an arrow between the first and second schematic saying “the larger the $k_{on}^{mask}$, the less time HER2 has to bind to trastuzumab, the more effective the mask is”, and an arrow between the second and third schematic saying “the lower the $k_{on}^{mask}$, the more time HER2 has to bind to trastuzumab, the less strongly the mask inactivates.

**Figure S12:** **High content imaging of trastuzumab (2 nM) by AlexaFluo488 secondary human antibody on breast cancer SK-BR-3 cells overexpressing HER2 after 0, 1 and 3 hours of incubation at 37 °C**, showing the binding of trastuzumab (in green), with or without the nucleus staining. No internalization was observed, which is consistent with data previously published(32).

**Figure S12 Alt text:** Figure showing microscopy images of the binding and internalization of trastuzumab to SK-BR-3 cells from 0 to 3 hours. Presence of trastuzumab, seeing in green, is only observed at the surface of the cells over this time.

**Figure S13:** **Flow cytometry raw data obtained for cells incubated with 400 nM protein, details of the gating strategy used to analyze and evaluate the binding to HER2 on SK-BR-3 cells.** In the first gate (P1), the singlets were selected (FSC-H: forward scatter height; FSC-A: forward scatter area). In the second gate (P2), the viable cells are selected (SSC-A: side scatter area). In the third gate (P3), the fluorescence on cells above a certain threshold was measured with the APC channel to detect Alexa Fluor 647. The median fluorescence intensity in the APC channel for the P2 population was reported.

**Figure S13 Alt text:** Figure showing the populations of cells iteratively selected on the flow cytometer: P1 (many points in diagonal), P2 (many points selected in a circle) and P3 (many points coming from the P2 selection). Two tables with the number of events and the percentage from the previous populations are shown.

**Figure S14:** *(A)* Flow cytometry (all data) showing some Hook effect at higher protein concentrations. The fitting shown is that reported in **Figure 3*E***. *(B)* The table shows the fitting for naked trastuzumab to which the TEV, Factor Xa or both enzymes were added.

**Figure S14 Alt text:** Graphs representing the binding (from fluorescence intensity) of intact and cleaved masked antibody (T, T-scFv40, T-scFv69, T-dAb. T-scFvGipg013 and NIP228) to HER2 on SK-BR-3 cells is shown as a function of antibody concentration, including high concentrations. The trends are the same as described in Figure 3, however here higher concentrations are shown, and the curves fall back down at the highest concentrations.

**Figure S15: Verification of the digestion of the masked antibodies (Fab) with TEV and Factor Xa protease by SDS-PAGE, in preparation for the SEC-MALS experiments.**

**Figure S15 Alt text:** Picture of a SDS-PAGE, where the position of the bands show the molecular weight of the protein, for masked Fab domains. The ladder is on the left hand-side. T-scFv40 Fab and T-scFv69 Fab show the main band at 75 kDa, T-dAb at 62.5 kDa, and when digested by TEV and Factor Xa, the main band migrated further (decreased further) and smaller bands corresponding to the digested fragment appeared.

**Figure S16:** **SEC-MALS traces for the different masked Fabs digested by the different enzymes.** The chromatograms are plotted on the left y-axis, and the MW (the horizontal line above or below the peaks of the chromatograms) on the right y-axis. (A) All intact masked antibodies. (B) T-scFv40. (C) T-scFv69. (D) T-dAb. (E) T-scFvGipg013.

**Figure S16 Alt text:** Figure made of five panels, showing chromatograms of differential refractive index. The chromatograms show peaks where the intact and digested Fabs eluted, and for each peak, the molecular weight detected by the multi angle scattering instrument is plotted. The different panels show the chromatograms and molecular weight for all intact (panel A) and digested constructs with TEV, Factor Xa or both enzymes, for T-scFv40 (panel B), T-scFv69 (panel C), T-dAb (panel D), T-scFvGipg013 (panel E).

**Figure S17: Normalized difference plot obtained by summing the significant D-incorporations relative to the reference, and subtracting the reference exchange to that of the different states**. T V_L_, T V_H_ were normalized with the same maxima. *(A)* Normalized differential deuterium uptake between trastuzumab variable heavy domain (V_H_) and T-scFv40, T-scFv40 + TEV, T-scFv40 + Factor Xa. *(B)* Normalized differential deuterium uptake between trastuzumab variable heavy domain (V_H_) and T-scFv69, T-scFv69 + TEV, T-scFv69 + Factor Xa, T-scFv69 + TEV + Factor Xa. *(C)* Normalized differential deuterium uptake between trastuzumab variable heavy domain (V_H_) and T-dAb, T-dAb + TEV. *(D)* Normalized differential deuterium uptake between trastuzumab variable heavy domain (V_H_) and T-scFvGipg013. *(E)* Normalized differential deuterium uptake between trastuzumab variable light domain (V_L_) and T-scFv40, T-scFv40 + TEV, T-scFv40 + Factor Xa. *(F)* Normalized differential deuterium uptake between trastuzumab variable light domain (V_L_) and T-scFv69, T-scFv69 + TEV, T-scFv69 + Factor Xa, T-scFv69 + TEV + Factor Xa. *(G)* Normalized differential deuterium uptake between trastuzumab variable light domain (V_L_) and T-dAb, T-dAb + TEV. *(H)* Normalized differential deuterium uptake between trastuzumab variable light domain (V_L_) and T-scFvGipg013.

**Figure S17 Alt text:** Figure made of eight panels, showing the deuterium incorporation of for each amino acid position, with the CDR regions highlighted, for the control, intact and digested masked antibody constructs: T-scFv40 (panels A, for the V_H_, and panel E, for the V_L_), T-scFv69 (panels B, for the V_H_, and panel F, for the V_L_), T-dAb (panels C, for the V_H_, and panel G, for the V_L_) and T-scFvGipg013 (only intact, panels D, for the V_H_, and panel H, for the V_L_).

**Figure S18: Schematic of the results of the HDX-MS experiments representing the paratope coverage on trastuzumab and the epitope coverage on the masks.** Crystal structure of the variable domains of trastuzumab observed from the top (1N8Z), scFv40, scFv69 (generated by homology modelling from 3AUV) and dAb (7PKL) observed from the bottom, colored by relative change in fractional deuterium exchange represented as a colour scale: reduced exchange (blue), no change (white), increased exchange (red), for trastuzumab interacting with the different masks scFv40, scFv69, dAb and scFvGipg013, with the linkers intact or digested by TEV and/or Factor Xa.

**Figure S18 Alt text:** Paratope of trastuzumab observed from the top and epitopes of the masks observed from the bottom. The figure is a representation of the protected regions on the paratope of trastuzumab and epitope of the masks, in blue, according to the HDX-MS results, for each of the masks (scFv40, scFv69, dAb and scFvGipg013), for intact and cleaved molecules. T-scFv69 has a smaller surface of protected regions compared to scFv40 and dAb, and the cleaved molecules still show blue regions on the paratope, although lighter.

**Figure S19: Peptide coverages of trastuzumab**. (A) Trastuzumab V_L_ (94.8 % coverage, 27 peptides. (B) Trastuzumab V_H_ (95.9% coverage, 51 peptides), obtained from a full-length IgG trastuzumab material. All the complementarity-determining regions (CDRs) were fully covered (highlighted in red), ensuring reliable information on the CDRs.

**Figure S19 Alt text:** Visual representation of the peptide coverage, where the amino acid sequence is written at the top, and bars corresponding to the peptides are plotted underneath, for the V_H_ and V_L_ of trastuzumab.

**Figure S20:** **HDX-MS uptake plots per peptide mapped on the light chain for the intact and digested masked antibodies** (T, T-dAb, T-dAb + TEV, T-scFv40, T-scFv40 + TEV, T-scFv40 + Factor Xa, T-scFv40 + TEV + Factor Xa, T-scFv69, T-scFv69 + TEV, T-scFv69 + Factor Xa, T-scFv69 + TEV + Factor Xa, T-scFvGipg013).

**Figure S20 Alt text:** Figure made of several plots, each corresponding to one peptide of the variable domain of the light chain. The graphs show the % deuterium incorporation over time, for T, T-dAb (intact and digested), T-scFv40 (intact and digested), T-scFv69 (intact and digested) and T-scFvGipg013 (intact).

**Figure S21: HDX-MS uptake plots per peptide mapped on the heavy chain for the intact and digested masked antibodies** (T, T-dAb, T-dAb + TEV, T-scFv40, T-scFv40 + TEV, T-scFv40 + Factor Xa, T-scFv40 + TEV + Factor Xa, T-scFv69, T-scFv69 + TEV, T-scFv69 + Factor Xa, T-scFv69 + TEV + Factor Xa, T-scFvGipg013).

**Figure S21 Alt text:** Figure made of several plots, each corresponding to one peptide of the variable domain of the heavy chain. The graphs show the % deuterium incorporation over time, for T, T-dAb (intact and digested), T-scFv40 (intact and digested), T-scFv69 (intact and digested) and T-scFvGipg013 (intact).

**Table S1: Comparison between the theoretical and experimental masses obtained in Figure S2 to S9**

|  |  | **Theoretical Average Mass (Da)** | **Experimental Mass (Da)** | **Error (ppm)** |
| --- | --- | --- | --- | --- |
| **T** | HC* | 50621.16 | 50626 | 95.68 |
|  | LC | 23438.79 | 23441 | 94.38 |
| **T-scFv40** | HC* | 50621.16 | 50619.86 | -25.55 |
|  | LC | 52012.68 | 52011.59 | -20.99 |
| **T-scFv69** | HC* | 50621.16 | 50619.72 | -28.48 |
|  | LC | 51944.65 | 51943.40 | -23.98 |
| **T-dAb** | HC* | 50621.16 | 50625 | 75.93 |
|  | LC | 39692.26 | 39696 | 94.28 |
| **T-scFvGipg013** | HC* | 50621.16 | 50619.71 | -28.56 |
|  | LC | 51931.56 | 51931.02 | -10.44 |
| **scFv40** | - | 27115.43 | 27116 | 21.02 |
| **scFv69** | - | 27047.40 | 27046.74 | -24.25 |
| **dAb** | - | 14572.77 | 14572.36 | -28.05 |

*The theoretical average mass for the heavy chain corresponds to the mass with the C-termal K clipped and the G0F glycosylation. The samples measured on the Synapt G2 were deconvoluted to the nearest dalton, whereas those measured on the Q Exactive had 4 significant number, 2 are shown in the table.

**Table S2: Sequences of the constructs investigated**

| scFv40 | | SELTQDPAVSVALGQTVRITCQGDSLRSYYASWYQQKPGQAPVLVIYGKNNRPSGIPDRFSGSSSGNTASLTITGAQA  EDEADYYCNSSDPDQLLVVFGGGTKLTVLGGGGGAGAENLYFQGGGGAGAEVQLLESGGGLVQPGGSLRLSCAASG  FTFSSYAMSWVRQAPGKGLEWVSAISGSGGSTYYADSVKGRFTISRDNSKNTLYLQMNSLRAEDTAVYYCAKNYQIHP  FDYWGQGTLVTVSSGGGGHHHHHHHH |
| --- | --- | --- |
| scFv69 | | SELTQDPAVSVALGQTVRITCQGDSLRSYYASWYQQKPGQAPVLVIYGKNNRPSGIPDRFSGSSSGNTASLTITGAQA  EDEADYYCNSSEPTPPRVVFGGGTKLTVLGGGGGAGAENLYFQGGGGAGAEVQLLESGGGLVQPGGSLRLSCAASG  FTFSSYAMSWVRQAPGKGLEWVSAISGSGGSTYYADSVKGRFTISRDNSKNTLYLQMNSLRAEDTAVYYCAKNVHIQ  PFDYWGQGTLVTVSSGGGGHHHHHHHH |
| dAb | | EVQLVESGGGLVQAGDSLTLSCAASGRTFSSVAMGWFRQAPGKERKFVANISWNGDSTYYTDSVKGRFTISRDNAK  NTVYLQMSSLKPEDTAVYYCAADVRWTGDGHRADYWGQGTQVTVSSGSGHHHHHHHH |
| T | **LC** | DIQMTQSPSSLSASVGDRVTITCRASQDVNTAVAWYQQKPGKAPKLLIYSASFLYSGVPS RFSGSRSGTDFTLTISSLQ  PEDFATYYCQQHYTTPPTFGQGTKVEIKRTVAAPSVFIFPPSDEQLKSGTASVVCLLNNFYPREAKVQWKVDNALQSG  NSQESVTEQDSKDSTYSLSSTLTLSKADYEKHKVYACEVTHQGLSSPVTKSFNRGEC |
|  | **HC** | EVQLVESGGGLVQPGGSLRLSCAASGFNIKDTYIHWVRQAPGKGLEWVARIYPTNGYTRYADSVKGRFTISADTSKN  TAYLQMNSLRAEDTAVYYCSRWGGDGFYAMDYWGQGTLVTVSSASTKGPSVFPLAPSSKSTSGGTAALGCLVKDYF  PEPVTVSWNSGALTSGVHTFPAVLQSSGLYSLSSVVTVPSSSLGTQTYICNVNHKPSNTKVDKRVEPKSCDKTHTCPPC  PAPELLGGPSVFLFPPKPKDTLMISRTPEVTCVVVDVSHEDPEVKFNWYVDGVEVHNAKTKPREEQYNSTYRVVSVLT  VLHQDWLNGKEYKCKVSNKALPAPIEKTISKAKGQPREPQVYTLPPSREEMTKNQVSLTCLVKGFYPSDIAVEWESNG  QPENNYKTTPPVLDSDGSFFLYSKLTVDKSRWQQGNVFSCSVMHEALHNHYTQKSLSLSPGK |
| T-scFv40* | **LC** | SELTQDPAVSVALGQTVRITCQGDSLRSYYASWYQQKPGQAPVLVIYGKNNRPSGIPDRFSGSSSGNTASLTITGAQAE  DEADYYCNSSDPDQLLVVFGGGTKLTVLGGGGGAGASGIEGRGGGGAGAEVQLLESGGGLVQPGGSLRLSCAASGFT  FSSYAMSWVRQAPGKGLEWVSAISGSGGSTYYADSVKGRFTISRDNSKNTLYLQMNSLRAEDTAVYYCAKNYQIHPFD  YWGQGTLVTVSSGSSGAGSGSAENLYFQGSGSAENLYFQGSGGADIQMTQSPSSLSASVGDRVTITCRASQDV  NTAVAWYQQKPGKAPKLLIYSASFLYSGVPSRFSGSRSGTDFTLTISSLQPEDFATYYCQQHYTTPPTFGQGTKVEIKRT  VAAPSVFIFPPSDEQLKSGTASVVCLLNNFYPREAKVQWKVDNALQSGNSQESVTEQDSKDSTYSLSSTLTLSKADYEK  HKVYACEVTHQGLSSPVTKSFNRGEC |
| T-scFv69* | **LC** | SELTQDPAVSVALGQTVRITCQGDSLRSYYASWYQQKPGQAPVLVIYGKNNRPSGIPDRFSGSSSGNTASLTITGAQAE  DEADYYCNSSEPTPPRVVFGGGTKLTVLGGGGGAGASGIEGRGGGGAGAEVQLLESGGGLVQPGGSLRLSCAASGFT  FSSYAMSWVRQAPGKGLEWVSAISGSGGSTYYADSVKGRFTISRDNSKNTLYLQMNSLRAEDTAVYYCAKNVHIQPF  DYWGQGTLVTVSSGSSGAGSGSAENLYFQGSGSAENLYFQGSGGADIQMTQSPSSLSASVGDRVTITCRASQDVNTA  VAWYQQKPGKAPKLLIYSASFLYSGVPSRFSGSRSGTDFTLTISSLQPEDFATYYCQQHYTTPPTFGQGTKVEIKRTVAAP  SVFIFPPSDEQLKSGTASVVCLLNNFYPREAKVQWKVDNALQSGNSQESVTEQDSKDSTYSLSSTLTLSKADYEKHKVYA  CEVTHQGLSSPVTKSFNRGEC |
| T-dAb* | **LC** | EVQLVESGGGLVQAGDSLTLSCAASGRTFSSVAMGWFRQAPGKERKFVANISWNGDSTYYTDSVKGRFTISRDNAKN  TVYLQMSSLKPEDTAVYYCAADVRWTGDGHRADYWGQGTQVTVSSGSSGAGSGSAENLYFQGSGSAENLYFQGSG  GADIQMTQSPSSLSASVGDRVTITCRASQDVNTAVAWYQQKPGKAPKLLIYSASFLYSGVPSRFSGSRSGTDFTLTISSL  QPEDFATYYCQQHYTTPPTFGQGTKVEIKRTVAAPSVFIFPPSDEQLKSGTASVVCLLNNFYPREAKVQWKVDNALQS  GNSQESVTEQDSKDSTYSLSSTLTLSKADYEKHKVYACEVTHQGLSSPVTKSFNRGEC |
| T-scFvGipg013* | **LC** | SYVLTQPPSASGTPGQRVAISCSGSNSNIGSNTVHWYQQLPGAAPKLLIYSNNQRPSGVPDRFSGSNSGTSASLAISRL  QSEDEADYYCAAWDDSLNGVVFGGGTKVTVLGGGGGAGASGIEGRGGGGAGAQVQLQQSGAEVKKPGSSVKVSC  KASGGTFSSYAISWVRQAPGQGLEWMGGIIPTFGTANYAQKFQGRVTITADESTSTAYMELSSLRSEDTAVYYCAQGP  IVGAPTDYWGKGTLVTVSSGSSGAGSGSAENLYFQGSGSAENLYFQGSGGADIQMTQSPSSLSASVGDRVTITCRASQ  DVNTAVAWYQQKPGKAPKLLIYSASFLYSGVPSRFSGSRSGTDFTLTISSLQPEDFATYYCQQHYTTPPTFGQGTKVEIK  RTVAAPSVFIFPPSDEQLKSGTASVVCLLNNFYPREAKVQWKVDNALQSGNSQESVTEQDSKDSTYSLSSTLTLSKADY  EKHKVYACEVTHQGLSSPVTKSFNRGEC |

* T-scFv40. T-scFv69. T-dAb and T-scFvGipg013 all have the same heavy chain as trastuzumab (T).

**Table S3: EC_50_ values for trastuzumab and digested controls**

|  | **EC_50_ (nM)** |
| --- | --- |
| T | 2.7 ± 0.6 |
| T +TEV | 4 ± 1 |
| T + Factor Xa | 3.6 ± 0.8 |
| T +TEV+ Factor Xa | 4 ± 1 |

**Table S4: Summary of HDX mass spectrometry experimental details**

| Dataset | T (10 µM)  T-scFv40 (10 µM)  T-scFv40 + TEV (10 µM)  T-scFv40 + Factor Xa (10 µM)  T-scFv40 + TEV + Factor Xa (10 µM)  T-scFv69 (10 µM)  T-scFv69 + TEV (10 µM)  T-scFv69 + Factor Xa (10 µM)  T-scFv69 + TEV + Factor Xa (10 µM)  T-dAb (10 µM)  T-dAb + TEV (10 µM)  T-scFvGipg013 (10 µM) |
| --- | --- |
| HDX reaction details | Equilibration in H_2_O: 10 mM potassium phosphate pH 7.5  Labelling in D_2_O: 10 mM potassium phopshate pD 7.5  Both were done at 20 °C  Quench buffer in H_2_O: 100 mM potassium phosphate, 0.5 M TCEP, 8 M urea pH 2.5 |
| HDX time course | 50 s, 500 s, 5000 s |
| HDX controls | 0 s |
| Number of peptides | T V_L_: 27  T V_H_: 51 |
| Sequence coverage | T V_L_: 94.8%  T V_H_: 95.9% |
| Average peptide length | T V_L_: 11.9 AA  T V_H_: 12.1 AA |
| Replicates | Technical replicates: 3 |
| Significant differences in HDX | The incorporation of deuterium per peptide is significant compared to the wild-type if p-value < 0.01 (t-test) |
| Repeatability | Average Student’s t-distribution 95% confidence interval for all peptides, all time points and states (#D error):  T V_L_: 0.339  T V_H_: 0.124 |

**Table S5: Data collection, processing, refinement and model statistics for the X-ray crystal structure of T-dAb Fab (PDB: 7PKL)**

| **pdbID** | **7PKL** |
| --- | --- |
| Description | Mechanistic understanding of antibody masking with anti-idiotypic antibody fragments |
| Crystallisaiton Conditions | 0.2M ammonium sulphate, 0.1M sodium acetate (pH 4.6) and 25% w/v peg 4000 |
| **Data Collection** |  |
| Wavelength | 0.97950 |
| Space Group | *P 21 21 2* |
| Unit Cell Dimensions | a=160.7420Å b=59.9650Å c=63.4990Å α=γ=β=90° |
| Resolution Range (Å) | 80.371-2.345 (2.592-2.345) |
| Number of Reflections | 105869 |
| Unique Reflections | 15991 |
| Multiplicity | 6.6 (5.7) |
| Completeness (%) | 93 |
| *<*I/sI*>* | 5.7 (1.4) |
| *R*_pim_ | 0.126 (0.546) |
| *CC_1/2_* | 0.979 (0.567) |
| *R_meas_ (all I+ & I-)* | 0.328 (1.366) |
| **Refinement** |  |
| Overall Wilson B (Å^2^) | 34.8 |
| R.m.s. deviations |  |
| Bond Lengths (Å) | 0.01 |
| Bond Angles (°) | 1.26 |
| No. of Atoms |  |
| Protein | 4224 |
| Het Atoms | 20 |
| Water | 253 |
| B Factors (Å^2^) |  |
| Protein | 35.00 |
| Water | 67.60 |
| *R*_work_ / *R*_free_ (%) | 18.0 / 26.5 |
| **Ramachandran Parameters** |  |
| Prefered (%) | 94.7 |
| Allowed (%) | 4.7 |
